# Requirement of hippocampal DG nNOS-CAPON dissociation for the anxiolytic and antidepressant effects of fluoxetine

**DOI:** 10.7150/thno.70370

**Published:** 2022-05-01

**Authors:** Hu-Jiang Shi, Dan-Lian Wu, Rong Chen, Na Li, Li-Juan Zhu

**Affiliations:** 1 Key Laboratory of Developmental Genes and Human Diseases, MOE, Department of Histology and Embryology, School of Medicine, Southeast University, Nanjing, Jiangsu 210009, PR China.; 2 Department of Pharmacy, The Affiliated Jiangyin Hospital, School of Medicine, Southeast University, Jiangyin, Jiangsu 214400, PR China.; 3 Shanghai Mental Health Center, Shanghai Jiao Tong University School of Medicine, Shanghai, 201108, China.

**Keywords:** anxiety, depression, fluoxetine, 5-HT1A receptor, nNOS-CAPON coupling

## Abstract

**Background:** Adult hippocampal neurogenesis and synaptic plasticity are necessary for the behavioral response to the selective serotonin reuptake inhibitor (SSRI) fluoxetine, but the molecular mechanisms underlying these effects are only partially understood.

**Methods:** Anxiety and depressive-like behaviors in mice were developed by chronic mild stress (CMS) or chronic corticosterone (CORT) treatment. Pharmacological and genetic approaches were used to investigate the role of the neuronal nitric oxide synthase (nNOS)-carboxy-terminal PDZ ligand of nNOS (CAPON) interaction in behavioral and neuroplasticity effects of serotoninergic system. Molecular biological and morphological studies were performed to examine the mechanisms underlying the behavioral effects of nNOS-CAPON interaction that modulated by 5-HT1A receptor (5-HT1AR).

**Results:** Fluoxetine prevented chronic stress-induced nNOS-CAPON upregulation and coupling in the dentate gyrus (DG), and promoting nNOS-CAPON association weakened the anxiolytic and antidepressant effects of fluoxetine in stressed mice. The chronic fluoxetine elevated 5-HT and 5HT1AR agonist 8-OH-DPAT decreased the expression and binding of nNOS with CAPON, whereas 5-HT1AR antagonist NAN-190 had the opposite effects. Importantly, augmenting nNOS-CAPON binding neutralized 8-OH-DPAT-upregulated spine density of DG granule cells and well-characterized synaptic-related proteins, including brain-derived neurotrophic factor (BDNF) and phosphorylation of extracellular signal regulated kinase (ERK), cAMP-response element binding protein (CREB), and synapsin in the DG and abolished the anxiolytic and antidepressant-like effects of 8-OH-DPAT. In contrast, dissociation of nNOS from CAPON rescued the effects of NAN-190 on behavior and neuroplasticity.

**Conclusion:** Taken together, our results indicated that fluoxetine modifies mood behaviors and hippocampal neuroplasticity by disrupting the nNOS-CAPON interaction that links postsynaptic 5-HT1AR activation.

## Introduction

Depression and anxiety are highly prevalent neuropsychiatric disorders that remain a major burden on society. Numerous reports have shown that anxiety and depression have a high comorbidity, and the co-occurrence rates are up to 60% in patients 1. Selective serotonin reuptake inhibitors (SSRIs), such as fluoxetine, are the most commonly prescribed drugs to treat depression and anxiety 2. SSRIs exert their therapeutic effects by inhibiting serotonin reuptake transporters (SERT) and then elevating extracellular concentrations of serotonin (5-hydroxtryptamine, 5-HT) 3. Although this elevation occurs rapidly, there is a delayed onset action of SSRIs after 3 to 4 weeks of continuous treatment. In addition, clinical reports indicate that the efficacy of SSRIs is incomplete and variable, as less than 60% of patients exhibit a significant response 4. Numerous studies have been conducted to explore the neurobiology of the antidepressant and anxiolytic actions of SSRIs, but the mechanisms by which SSRIs influence depressive and anxious behaviors have only been partially elucidated.

The 5-HT system of mammalian brain originates from the raphe, where 5-HT neurons are clustered into nine nuclei on a rostrocaudal axis 5. The rostral subdivision 5-HT neurons project primarily to the forebrain, where their terminals widely distributed densely innervates almost all regions 6. The hippocampus, one of the important components of limbic system, is densely innervated by serotonergic projections and implicated in the neuropathology of psychiatric disorders 7. Considerable evidence links hippocampal neurogenesis and synaptogenesis to the antidepressant and anxiolytic actions of SSRIs 2, 4, 8. Better understand how SSRIs modulate 5-HTergic neuronal circuitry leading to behavioral changes is critical for the development of novel faster-acting antidepressants and anxiolytics.

A diverse range of evidences support the involvement of the 5-HT1A receptor (5-HT1AR) subtype in the regulation of anxiety and depression and is implicated in the behavior and neuroplasticity response to SSRIs 9-11. There are two distinct populations of 5-HT1AR: somatodendritic autoreceptors on the raphe nuclei neurons and postsynaptic heteroreceptors in regions innervated by serotonergic fibers 10. Expression of postsynaptic 5-HT1A heteroreceptors in the hippocampus exhibits a unique pattern in which it was more prominent in the ventral dentate gyrus (DG) subfield 12. Recently, Samuels and colleagues reported that 5-HT1A heteroreceptors on mature granule cells of hippocampal DG are sufficient to mediate the effects of fluoxetine on behaviors and neurogenesis 11. There was also evidence that fluoxetine specifically promoted proliferation of neural stem cells (NSCs) in the ventral hippocampus, a subregion more associated with emotional regulation, but not in the dorsal hippocampus, and this effect of fluoxetine was mediated by 5-HT1AR 13. Meanwhile, hippocampal synaptic dysfunctions with neuronal atrophy and synaptic structural and transmission impairment were demonstrated in depressive and anxiety disorders, which were reversed upon treatment with SSRIs 14, 15. Chronic fluoxetine treatment stimulated the development of dendritic arborization, facilitated the maturation of immature granule cells, enhanced the survival of young neurons, stimulated the functional integration of young neurons into the local hippocampal circuit and enhanced the long-term synaptic plasticity of DG granule cells 2. Additionally, we previously reported that 5-HT1AR modulates hippocampal synaptogenesis, including dendritic branching and dendritic spine density of DG granule cells, via cAMP-response element binding protein (CREB) activation 16. Although the serotoninergic system has distinctive neuroplastic capabilities, including neurogenic and synaptogenic effects, the molecular mechanisms underlying the effects of SSRIs remain unclear. More importantly, approaches targeting specific 5-HT1ARs or its downstream pathways, rather than elevating serotonin like SSRIs, may contribute to improved treatment strategies 11.

Our previous studies demonstrated that hippocampal neuronal nitric oxide synthase (nNOS)-carboxy-terminal PDZ ligand of nNOS (CAPON, also known as NOS1AP) coupling represents a therapeutic target for developing new anxiolytics 17. Augmenting the nNOS-CAPON interaction produced anxiogenic effects, while dissociating CAPON from nNOS reversed chronic stress-induced anxiogenic behaviors. Moreover, hippocampal synaptic plasticity and Dexras1-p-ERK signaling play crucial roles in nNOS-CAPON binding-induced regulation of anxiety behaviors. The current study was conducted to explore whether fluoxetine exerts behavioral and neuroplasticity actions by regulating hippocampal DG nNOS-CAPON interaction. We found that fluoxetine treatment prevented the chronic stress-induced association of nNOS with CAPON, while enhancement of the nNOS-CAPON association eliminated the anxiolytic and antidepressant effects of fluoxetine. In particular, our results showed that the increase in serotonin levels induced by SSRIs activates 5-HT1A receptors, and enhances hippocampal neurogenesis and dendritic spine remodeling, thus regulating anxiety and depression behaviors by affecting the nNOS-CAPON interaction. Several studies have linked the role of ERK-CREB-BDNF signaling to the serotonergic system in hippocampal neurogenesis, dendritic remodeling, and synaptic plasticity 10, 18. We found that dissociation of nNOS from CAPON prevents NAN-190-induced downregulation of phosphorylation of extracellular signal regulated kinase (ERK), CREB and synapsin and the expression of brain-derived neurotrophic factor (BDNF), while augmentation of nNOS-CAPON binding reverses the effect of 8-OH-DPAT on ERK-CREB-BDNF-synapsin signaling. Together, our data highlighted the important role of nNOS-CAPON coupling in the behavioral and neuroplasticity effects of fluoxetine and revealed new molecular substrates implicated in the responses to fluoxetine.

## Materials and methods

### Animals

Young adult (6-7 weeks, 22-28 g) ICR male mice and newborn (postnatal d P0-P1) ICR mice (RRID: IMSR_RBRC05979) (from Model Animal Research Center of Nanjing University, Nanjing, China) were used in this study. All procedures involving the use of animals were approved by the Institutional Animal Care and Use Committee of Southeast University (IACUC-20191025011). Animals were euthanized by administering isoflurane as an anaesthetic followed by decapitation as a confirmation method.

### Drugs and systemic injections

Fluoxetine (Cat# F132), (±) -8-Hydroxy-2-(dipropylamino) tetralin hydrobromide (8-OH-DPAT, Cat# H8520) and serotonin hydrochloride (5-HT, Cat# H9523) were purchased from Sigma-Aldrich (St Louis, MO, USA) and were dissolved in saline. 1-(2-Methoxyphenyl)-4-[4-(2-phthalimido) butyl] piperazine hydrobromide (NAN-190, Cat# 0553) was purchased from TOCRIS bioscience (Bristol, UK) and dissolved in saline. Betulinic acid (Cat# HY-10529) was purchased from MedChemExpress (New Jersey, USA) and dissolved in PBS. Fluoxetine (10 mg/kg/d), 8-OH-DPAT (0.1 mg/kg/d), and NAN-190 (0.3 mg/kg/d) were intraperitoneally injected for 28 days. 5-HT (10 mg/kg/d) was intraperitoneally injected for 7 days.

### Intrahippocampal infusions

Corticosterone (CORT, Cat# 27840, Sigma-Aldrich, St Louis, MO, USA) was dissolved in 0.1% DMSO and was delivered into the DG of the hippocampus by microinjection as described previously 19. The osmotic pump (Alzet®, Cat# 2004, 200 μL) containing CORT solution were placed subcutaneously in the back of mice until the mice were killed, and brain infusion cannula connected to the pump were positioned at the following coordinates: 2.3 mm posterior to bregma, 1.3 mm lateral to the midline, and 2.0 mm below dura. The infusion rate of the osmotic pump was 0.25 μL/h. We separated the cannula connected to the osmotic pump from the catheter to stop pumping into the CORT 1 day before behavioral tests.

### Chronic mild stress and behavioral tests

The chronic mile stress (CMS) procedure was designed as described in our previous studies 20. It was consisted of a variety of mild stressors, including restraint, forced swimming, food/water deprivation, tilted cage (45° from horizontal), housed in wet sawdust, and reversal of the light/dark cycle. CMS was conducted for 4 weeks for all stress groups.

The behavioral tests were performed as described in our previous study 21-24, including the open-field test (OFT), novelty-suppressed feeding test (NSFT), as well as the elevated zero maze (O-maze), forced swimming test (FST) and tail suspension test (TST).

For OFT, each mouse was placed in a square chamber (40×40 cm). The movement of the mice was recorded using a digital camera. The recorded video file was further analyzed using EthoVision 11.5 (Noldus). The data were collected over 30 min, and the number of entries into, the overall time spent in, and the mean distance to the center of the arena (20×20 cm imaginary square) were measured during the first 5 min. The automatic movement ability was analyzed for the first 5 min.

The NSF test was carried out over a 5-min period. The testing apparatus consisted of a plastic box (50 × 50 × 20 cm). Mice were given NSF test after being deprived of food for 24 hours. The latency to feed regular chow in a novel environment in 5 min has been used as an index of anxiety-related behaviors. The latency to feed in 5 min and the amount of food consumed by the mouse in 15 min in the home cage were used as a control for appetite changes as a possible confounding factor.

For O-maze test, the equipment was a 100 cm in height, 45 cm in outer diameter and 6 cm wide ring with two open arms and two closed arms. Mice were placed at the open-closed boundary facing the open side. The time spent in the open and closed arms and the total numbers of entries into the open arm were recorded for 10 min. The number of entries into and the time spent in the open and closed arms during the first 5 min of the test were analyzed using EthoVision 11.5 (Noldus). The time spent in the open arms was used as an index of anxiety-related behavior and a total number of entries into the total (open + enclosed) arms as a general activity.

For the TST, mice were suspended from the hook of a TST box 60 cm above the surface of a table using adhesive tape placed 1 cm from the tip of the tail. After 1 min of acclimatization, the immobility duration was recorded by a camera for 6 min and was analyzed using PhenoScan Series (Clever Syn. Inc). Mice were considered immobile only when they hung passively and were completely motionless.

For FST, mice were placed individually in a glass cylinder (20 cm height, 17 cm diameter) filled with water to a depth of 10 cm at 23℃ to 25℃ and swam for 5 min. The water depth was set to prevent the animals from touching the bottom with their tails or hind limbs. The animal behaviors were videotaped from the side. The immobile time during the 5-min test was analyzed using PhenoScan Series (Clever Syn. Inc). The immobile time was defined as the time during which the animals remained floating or motionless with only movements necessary for maintaining balance in the water.

After each trial, the plate was cleaned with 70% ethyl alcohol (EtOH). The behavioral tests were initiated approximately 24 hours after the last injection of the drugs. During the experiments and analysis, the investigators were blinded to the treatment group. The mice were identified using earmarks, and their numbers were given only after the behavioral experiments and analysis were completed.

### Culture of neurons

Primary neurons were cultured as reported previously 23. The planting density was 2 × 10^4^ cells/cm^2^ for morphological analysis and 1 × 10^5^ cells/cm^2^ for biochemical detection.

### Isolation of synaptosomes and immunoblotting

Synaptosomes were prepared as described previously with modification 25. Briefly, the tissue was rapidly removed and homogenized in preparation buffer (0.32 M sucrose, 1 mM NaHCO_3_, 1 mM MgCl_2_, 0.5 mM CaCl_2_, 1 mM PMSF and protease inhibitors) with Tissuelyser at 4 °C. The homogenate was centrifuged at 500× g for 2 min to collect the supernatant (S1). S1 fraction was centrifuged at 10,100× g for 10 min to obtain the pellet (P2) and supernatant (S2). The P2 fractions were re-suspended in 0.32 M sucrose, which were then layered onto 0.8 M sucrose. After being centrifuged at 9,100× g for 15 min in a swinging bucket rotor, synaptosomes were collected from 0.8 M sucrose layer. All procedures were performed at 4 °C, and buffers or solutions contained protease inhibitors (Cat# 04693132001; Roche) and phosphatase inhibitors (Cat# 04906837001; Roche). Samples were processed for SDS-PAGE, transferred, probed with antibodies, and visualized with enhanced chemiluminescence.

### Western blot analysis

Western blot analysis of samples from cultured neurons and hippocampal tissues from animals was performed as described previously 20, 23. Briefly, the samples were lysed with RIPA (Cat# P0013C, Beyotime Biotechnology, Shanghai, China). Protein concentrations were determined with the BCA Protein Assay Kit (Cat#23227, Thermo Fisher Scientific, Waltham, MA, USA). The total proteins were loaded on 8%-12% SDS-PAGE gels for electrophoresis followed by transferring to the PVDF membrane (Cat# IPVH00010, Millipore Bioscience Research Reagents, Temecula, CA, USA). Then the membranes were blocked by 5% skimmed milk, and the primary antibodies were incubated overnight at 4 °C. The primary antibodies used were as follows: mouse anti-nNOS (1:1000; Cat# 610308, RRID: AB_397700, BD Transduction Laboratories, Franklin Lakes, NJ), rabbit anti-nNOS (1:2000; Cat# AB5380, RRID: AB_91824, Millipore Bioscience Research Reagents, Temecula, CA, USA), rabbit anti-CAPON (1:10000; Cat# ab190686, RRID: AB_2713895, Abcam, MA, USA), rabbit anti-synapsin Ι (1:24000; Cat# AB1543P, RRID:AB_90757, Millipore Bioscience Research Reagents), rabbit anti-phospho-synapsin Ι-Ser 9 (1:30000; Cat# ab76260, RRID:AB_1524461, Abcam), rabbit anti-phospho-synapsin Ι-Ser 62,67 (1:20000; Cat# AB9848, RRID:AB_673006, Millipore Bioscience Research Reagents), mouse anti-BDNF (1:1000; Cat# ab203573, RRID:AB_2631315, Abcam), rabbit anti-CREB (1:2000; Cat# 9197, RRID:AB_331277, Cell Signaling Technology, Beverly, MA, USA), rabbit anti-phospho-CREB-Ser133 (1:1000; Cat# 9198, RRID:AB_2561044, Cell Signaling Technology), rabbit anti-ERK (1:2000; Cat# 9102, RRID:RRID:AB_330744, Cell Signaling Technology), rabbit anti-phospho-ERK-Thr202/Tyr204 (1:1000; Cat# 9101, RRID:AB_331646, Cell Signaling Technology), rabbit anti-p65 (1:1000; Cat# ab16502, RRID:AB_443394; Abcam), rabbit anti-p50 (1:500; Cat# ab7971 RRID:AB_306185; Abcam). Internal control was performed using rabbit anti-β-actin antibody (1:20000; Cat# ab8227, RRID: AB_2305186, Abcam). Appropriate horseradish peroxidase-linked secondary antibodies (Cat# GAR007, RRID:AB_2827833; Cat# GAM0072, RRID:AB_2827834, MultiSicences) were used for detection by enhanced chemiluminescence (Cat# 34577, Thermo Fisher Scientific, Waltham, MA). In statistical analysis, chemifluorescence was detected by Tanon-5200 Imaging system (Tanon Science & Technology, Shanghai, China). Quantitation of band density was conducted on analysis within the linear range and by a blinding approach. The intensity of each band was normalized to the intensity of internal standard (such as β-actin) band. Then all values (control and test) were normalized to the mean value of the experimental control group to set the Y-axis so the control group value was 1. Each sample was repeated more than three times.

### Coimmunoprecipitation (CO-IP)

The coimmunoprecipitation assay was performed as described previously^17^, ^20^, ^23^. Briefly, the hippocampal DG of mice was homogenized in ice-cold lysis buffer A (Cat# R0278; Sigma-Aldrich) for 30 min, and then centrifuged at 20 000 g for 15 min. The supernatant was pre-incubated for 1 h at 4 °C with 0.02 mL of protein G-Sepharose beads (Cat# P4691; Sigma-Aldrich). It was then centrifuged to remove proteins that adhered non-specifically to the beads and to obtain the target supernatant for the following IP experiment. Protein G-Sepharose beads were incubated with an antibody (mouse antibody to nNOS, Cat# 610308, RRID: AB_397700, 5 μg; BD Transduction Laboratories) for 3-4 h. The antibody-conjugated protein G-Sepharose beads were incubated with the target supernatant overnight. The complexes were isolated using the centrifuge, washed four times with 0.05 M HEPES buffer pH 7.1 containing 0.15% Triton X-100 and 0.15 M NaCl. Bound proteins were eluted by heating the solution at 100 °C in loading buffer for 10 min. The analysis was conducted by immunoblotting.

### Immunofluorescence

The details of immunofluorescence for brain sections and cultured neurons have been described 26. In brief, for immunohistochemistry, mice were transcardially perfused with 0.9% NaCl followed by 4% PFA under anesthesia (ketamine), and their brains were cryoprotected in 30% sucrose and cryosectioned at 40 μm thickness using a cryostat (CM 1950, Leica, Nubloch, Germany). Slices were blocked in PBS containing 3% normal goat serum, 0.3% (w/v) Triton X-100, and 0.1% BSA at room temperature for 1 h, followed by incubation in primary antibody at 4 °C overnight. The primary antibodies were used as follows: rat anti-Brdu (1:200; Cat# OBT0030G, RRID:AB_609567, AbD Serotec), mouse anti-NeuN (1;1000; Cat# MAB377, RRID:AB_2298772, Millipore). Secondary antibodies used were goat anti-Rat Cy3 (1:200; Cat#112-165-003, RRID: AB_2338240, Jackson ImmunoResearch Laboratories) and goat anti-Mouse Alexa-488 (1:400; Cat# 115-545-146, RRID: AB_2307324, Jackson ImmunoResearch Laboratories). Images were captured with a confocal laser-scanning microscope (FV3000, Olympus, Tokyo, Japan) at identical settings for each of the conditions and were analyzed using ImageJ by an experimenter blind to treatment groups. The analysis was conducted on every 6th section in a series of 40 μm sections through dentate gyrus. The total number of BrdU+ cells per DG was determined by the mean value of the counts from sampled sections and multiplied by the total number of sections, which was regarded as the final value of one sample.

Neurons at 7 DIV were treated with 10 μM CORT alone or in combination with 0.1 μM fluoxetine or vehicle for 3 days. At 10 DIV, neurons were fixed and incubated with primary antibodies overnight at 4 °C, followed by incubation with the secondary antibodies for 2 h at room temperature. The primary antibodies used were as follows: rabbit anti-p65 (1:1000; Cat #ab16502, RRID: AB_443394; Abcam) and chicken anti-MAP2 (1:5000; Cat# NB300-213, RRID: AB_2138178; Novus, CO, USA). The secondary antibodies used were goat anti-rabbit Cy3 (1:200; Cat#111-165-003, RRID: AB_2338000; Jackson ImmunoResearch Laboratories) and goat anti-chicken Alexa-488 (1:400; Cat# 103-545-155, RRID: AB_2337390; Jackson ImmunoResearch Laboratories). Finally, the cultures were counter-stained with DAPI (Cat# D9564; Sigma-Aldrich) to label the nuclei. Images were captured with a confocal laser-scanning microscope (A1 HD25, Nikon, Tokyo, Japan) at identical settings for each of the conditions.

### Duolink proximity ligation assay (PLA)

The PLA was performed on cultured neurons with a Duolink in situ detection kit as described previously 20. Primary neurons were fixed at 10 DIV with 4% PFA for 15 min at room temperature and 10 min at 4 °C, followed with methanol for 1 min at -20 °C, and then permeabilized with 0.1% Triton X-100 in PBS for 5 min. Cells were incubated with Duolink blocking solution for 30 min at 37 °C, followed by incubation with monoclonal mouse nNOS (1:50; Cat# 610308, RRID: AB_397700; BD Transduction Laboratories), rabbit CAPON (1:200; Cat# ab190686, RRID: AB_2713895; Abcam) and chicken β3-tubulin (1:500; Cat# 302306, RRID: AB_2620048; Synaptic Systems, Göttingen, Germany) primary antibodies diluted in Duolink Antibody diluent overnight at 4°C. The ligation assay was then conducted following the instructions from the manufacturer (Cat# DUO92101, Sigma-Aldrich). β3-tubulin was detected using goat anti-chicken Alexa-488 (1:400; Cat# 103-545-155, RRID: AB_2337390; Jackson ImmunoResearch Laboratories). Images were captured using a confocal laser-scanning microscope (LSM700, Carl Zeiss, Oberkochen, Germany) at identical settings for each of the conditions. The fluorescence intensity of PLA signals was quantified with the ImageJ software (http://imagej.net).

### High-performance liquid chromatography with fluorescence detector (HPLC-FLD)

For determination of 5-HT and 5-HIAA, the brain was immediately extracted and the hippocampus was specifically dissected. The hippocampus was dissected, weighted, and then homogenized in 0.4M HClO_4_ buffer with Tissuelyser at a concentration of 0.2g of tissue per ml. Samples were centrifuged at 12,500× g for 20 min, and then centrifugated at 18000× g for 10 min at 4 °C to collect the supernatant. The resulting supernatant was filtered through 0.22μm syringe filter. Finally, one aliquot corresponding to 2 mg of tissue was assessed by Agilent 1260 high-performance liquid chromatography with fluorescence detector (Agilent technologies, Santa Clara, CA, USA). Chromatographic separation was achieved on an Altimate C18 (4.6×250 mm, 5 μm) column with the column temperature set at room temperature on Agilent 1260 system. Elution was performed with mobile phase A (0.1% formic acid-methanol) and B (0.1% formic acid) at 1 mL/min flow rate and the injection volume was 10 μL, following the gradient as follows: 10% A (0-5 min) → 60% A (5-7 min) → 90% A (3 min) → 90% A (10-10.5 min) → 10% A (7.5 min). Quantification of the sample peaks was done by comparing peak areas with those of standards. A calibration curve was performed daily within a concentration range of 0, 5, 10, 25, 100, 250, 500 μg/L of each standard per injection. The concentrations of 5-HT and 5-HIAA in the tissue samples were calculated by extrapolation of the samples' peak heights to the calibration curve.

### Golgi-Cox staining

Golgi-Cox staining was performed as described previously 17,20. Fresh, non-perfused brains were used for Golgi-Cox staining with FD Rapid GolgiStain Kit (Cat# PK401; FD NeuroTechnologies, MD, USA) according to the user manual, and the brains were cut into 100-μm coronal sections using a vibratome (VT1000s; Leica). To calculate the spine density of Golgi-stained neurons in the DG, a length of a dendrite was traced, the exact length of the dendritic segment was calculated, and the number of spines along the dendritic segment was counted. Ten random neurons in each sample were measured, and the average was regarded as the final value for one sample.

### Recombinant virus production and their stereotaxic injection

Adeno-associated virus (AAV)-cytomegalovirus (CMV)-CAPON-125C-GFP (green fluorescent protein)-3Flag was generated by GeneChem Co, Ltd, Shanghai, China. The coding sequence of mouse CAPON-125C was amplified by RT-PCR. The primers were as follows. Forward: 5'-GAG GAT CCC CGG GTA CCG GTC GCC ACC ATG ATC ACC TTC CGT TCA GG-3'; reverse: 5'-TCA CCA TGG GGC GAC CGG CAC GGC GAT CTC ATC ATC C-3'. CAPON-125C genes were sub-cloned into pAAV.CMV.EGFP.3Flag plasmids to produce pAAV.CMV.CAPON-125C.EGFP.3FLAG. AAV-CMV-CAPON-125C-GFP-3Flag was produced by transfection of AAV-293 cells with the above corresponding plasmids, AAV helper plasmid (pHelper) and AAV Rep/Cap expression plasmid (pAAV-RC). Viral particles were purified by an iodixanol step-gradient ultracentrifugation method. The genomic titer was 1.2-1.5 × 1013 genomic copies per ml determined by quantitative PCR.

To produce neuron-specific rAAV, CAPON-L gene were sub-cloned into pAAV.SYN.EGFP.3FLAG plasmids to produce pAAV.SYN.CAPON-L.EGFP.3FLAG (Neuron Biotech Co., Ltd, Shanghai, China), which has a neuron-specific promoter, the human synapsin I promoter (hSyn), the 469-bp human sequence chrX:47,364,154-47,364,622 27. The coding sequence of mouse CAPON-L was amplified by RT-PCR. The primers were as follows. Forward: 5'-GAG GAT CCC CGG GTA CCG GTC GCC ACC ATG CCC AGC AAA ACC AAG TAC AAC CTT GTG G-3', reverse: 5'-TCA CCA TGG TGG CGA CCG GCA CGG CGA TCT CAT CAT C-3; rAAV AAV-CAPON-L-GFP was produced by transfection of AAV-293 cells with pAAV.SYN.CAPON-L.EGFP.3FLAG, AAV helper plasmid (pAAV Helper) and AAV Rep/Cap expression plasmid. Viral particles were purified by an iodixanol step-gradient ultracentrifugation method. The genomic titer was 2.5-3.5 × 1012 genomic copies per ml determined by quantitative PCR.

The animals were placed in the stereotaxic devices (RWD, 68030) and kept anesthetized with isoflurane (1%) during virus injection. The skull above the targeted areas was opened with a cranial drill carefully. Injections were conducted with a syringe pump (KDS LEGATO 130) connected to a Hamilton syringe (Hamilton 87925). The virus was delivered (1 nL/s, 1 μL) into the hippocampal DG at coordinates (mm): 2.3 mm posterior to bregma, 1.3 mm lateral to the midline, and 2.0 mm below the dura.

### Statistical analysis

Data are presented as the mean ± SEM. The sample size was predetermined by our prior experience 17, 22, 23, 28. The Shapiro-Wilk test was carried out to assess the normality of data in the animal behavioral tests and the normalized data from the biochemical tests. If data were found to be normally distributed, then a one-way ANOVA with post-test of Tukey's multiple comparisons tests or General Linear Model was carried out, as specified (only if F in ANOVA achieved p < 0.05 and there was no significant variance inhomogeneity). If data were found to be normally distributed, but with significant variance inhomogeneity, then a Welch's ANOVA followed by Dunnett's T3 multiple comparisons test was carried out to test significance (only if F in ANOVA achieved p < 0.05). If data were found to not be normally distributed, then a Kruskal-Wallis test with a Dunn's multiple comparison post-test was carried out to test for significance. The above statistical analysis was conducted using GraphPad Prism version 8.3.0 (La Jolla, CA, USA) or IBM SPSS version 26.0 (Armonk, NY,USA). A p-value of < 0.05 was considered statistically significant. Investigators were blinded to the treatment groups when assessing the outcomes. No values were excluded in the data analysis and presentation.

## Results

### Fluoxetine attenuated chronic stress-induced behavioral modification through nNOS-CAPON dissociation

**Figure 1:**
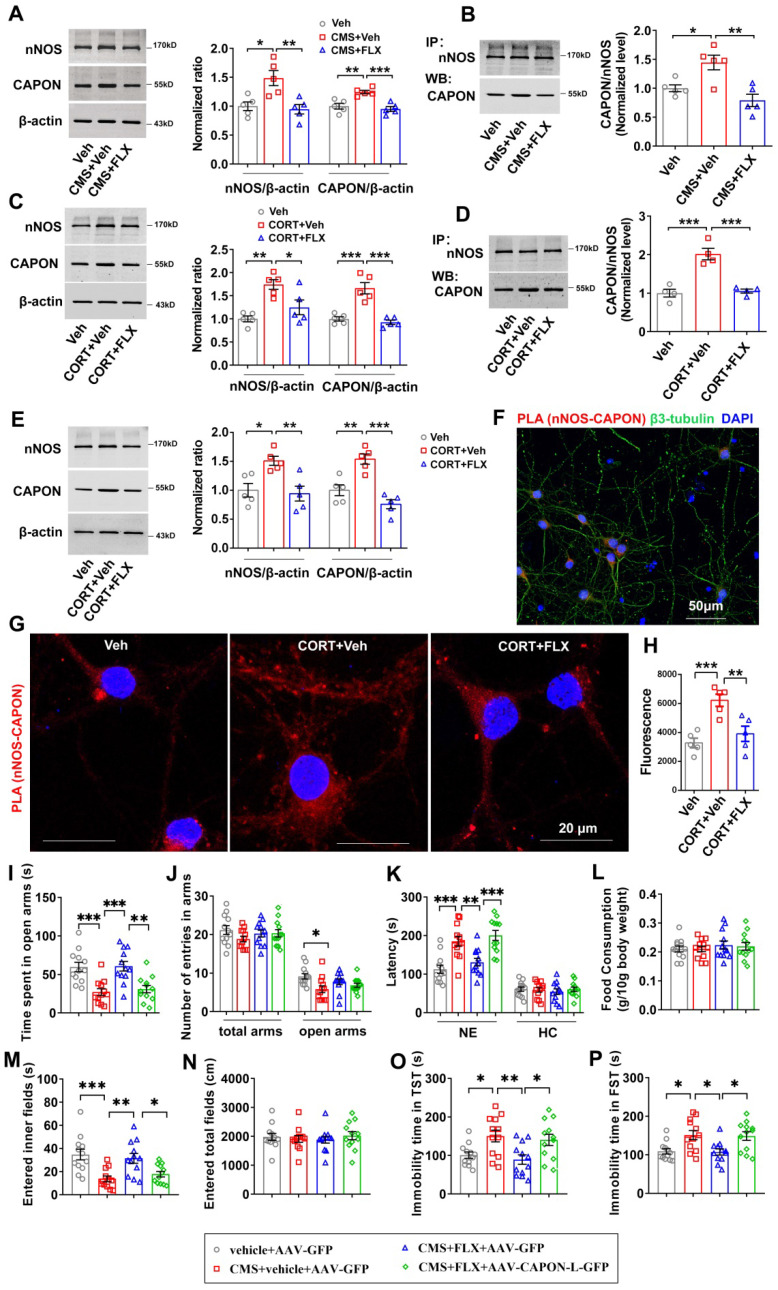
** Fluoxetine attenuated chronic stress-induced behavioral modification through nNOS-CAPON dissociation.** (**A-B**) The adult male ICR mice were treated with FLX (10 mg/kg/d) or its vehicle by intraperitoneal administration for 28 consecutive d and exposed to CMS. (**A**) Representative immunoblots (left) and bar graph (right) showing nNOS and CAPON in the hippocampal DG of mice treated with FLX and exposed to CMS (n = 5, for nNOS: *F*(2,12) = 9.083; CMS + vehicle versus vehicle: *p = 0.0115; CMS + FLX versus CMS + vehicle: **p = 0.0059; for CAPON: *F*(2,12) = 14.26; CMS + vehicle versus vehicle: **p = 0.0035; CMS + FLX versus CMS + vehicle: ***p = 0.0009). **(B)** FLX treatment reduced the amounts of nNOS-CAPON complex (presented as the ratio of CAPON to nNOS) in the hippocampal DG of mice exposed to CMS for 28 days (n = 5, *F*(2,12) = 10.83; CMS + vehicle versus vehicle: *p = 0.0231; CMS + FLX versus CMS + vehicle: **p = 0.0018). (**C-D**) The adult male ICR mice were treated with CORT (10 µM) alone or in combination with FLX (10 mg/kg/d) or its vehicle by intraperitoneal administration for 28 consecutive d. (**C**) Representative immunoblots (left) and bar graph (right) showing nNOS and CAPON in the hippocampal DG of mice treated with FLX and exposed to CORT (n = 5, for nNOS: *F*(2,12) = 10.65; CORT versus vehicle: **p = 0.0018; CORT + FLX versus CORT: *p = 0.0271; for CAPON: *F*(2,12) = 25.96; CORT versus vehicle: ***p = 0.0002; CORT + FLX versus CORT: ***p < 0.0001). **(D)** FLX treatment reduced the amounts of nNOS-CAPON complex in the hippocampal DG of mice exposed to CORT for 28 days (n = 4, *F*(2,9) = 27.83; CORT versus vehicle: ***p = 0.0003; CORT + FLX versus CORT: ***p = 0.0004). (**E-H**) The cultured neurons treated with 10 μM CORT alone or in combination with 0.1 µM FLX or their vehicle for 72 hours. **(E)** Representative immunoblots (left) and bar graph (right) showing nNOS and CAPON in the cultured neurons treated with FLX and exposed to CORT (n = 5, for nNOS: *F*(2,12) = 8.023; CORT versus vehicle: *p = 0.0172; CORT + FLX versus CORT: **p = 0.0088; for CAPON: *F*(2,12) = 15.38; CORT versus vehicle: **p = 0.007; CORT + FLX versus CORT: ***p = 0.0004). (**F-G**) Representative confocal images of in situ proximity ligation assay (PLA) between nNOS and CAPON (red) in primary neurons Maximum intensity projections of a confocal z-stack including a whole cell were performed to observe the maximum amount of PLA puncta. The neurons were counter-stained with DAPI (blue). Scale bar: 50μm **(F)**, 20μm **(G)**. **(H)** The quantification of PLA signals (nNOS-CAPON coupling) in primary neurons (n = 5 independent experiments, *F*(2, 12) = 12.87; CORT versus vehicle: ***p < 0.001; CORT + FLX versus CORT: **p = 0.0051). (**I-P**) The adult mice treated with intra-hippocampal microinjection of AAV-CAPON-L-GFP or AAV-GFP in combination with FLX (10 mg/kg/d) or its vehicle by intraperitoneal administration for 28 consecutive d and exposed to CMS. (**I**) The time spent in open arms (*F*(3,44) = 10.94, CMS + vehicle + AAV-GFP versus vehicle + AAV-GFP: ***p = 0.0007; CMS+ FLX + AAV-GFP versus CMS + vehicle + AAV-GFP: ***p = 0.0005; CMS+ FLX + AAV-CAPON-L-GFP versus CMS+ FLX + AAV-GFP: **p = 0.0021), (**J**) number of entries in the arms (for total arms: *F*(3,44) = 1.056, *p* = 0.3772; for open arms:* F*(3,44) = 3.849, CMS + vehicle + AAV-GFP versus vehicle + AAV-GFP: *p = 0.011) in the O maze test. (**K**) The latency to feed in a novel environment (*F*(3,44) = 13.23, CMS + vehicle + AAV-GFP versus vehicle + AAV-GFP: ***p = 0.0004; CMS+ FLX + AAV-GFP versus CMS + vehicle + AAV-GFP: **p = 0.0098; CMS+ FLX + AAV-CAPON-L-GFP versus CMS+ FLX + AAV-GFP: ***p = 0.0006) and in the home cage (*F*(3,44) = 0.2426, *p* = 0.8661) and (**L**) food consumption in the home cage (*F*(3,44) = 0.3042, *p* = 0.8222) in the NSFT in adult mice. (M) The time of entered inner fields (*F*(3,44) = 8.212, CMS + vehicle + AAV-GFP versus vehicle + AAV-GFP: ***p = 0.0008; CMS+ FLX + AAV-GFP versus CMS + vehicle + AAV-GFP: **p = 0.0057; CMS+ FLX + AAV-CAPON-L-GFP versus CMS+ FLX + AAV-GFP: *p = 0.0458) and (**N**) the total distance traveled (*F*(3,44) = 0.2639, *p* = 0.851) in the OFT. (**O**) The immobility time in the TST (*F*(3,44) = 5.323, CMS + vehicle + AAV-GFP versus vehicle + AAV-GFP: *p = 0.0445; CMS+ FLX + AAV-GFP versus CMS + vehicle + AAV-GFP: **p = 0.0087; CMS+ FLX + AAV-CAPON-L-GFP versus CMS+ FLX + AAV-GFP: *p = 0.036) and (**P**) FST (*F*(3,44) = 6.252, CMS + vehicle + AAV-GFP versus vehicle + AAV-GFP: *p = 0.0176; CMS+ FLX + AAV-GFP versus CMS + vehicle + AAV-GFP: *p = 0.0128; CMS+ FLX + AAV-CAPON-L-GFP versus CMS+ FLX + AAV-GFP: *p = 0.0207) of the adult mice. Mean ± SEM, *P < 0.05, **P < 0.01, ***P < 0.001, one-way ANOVA followed by Tukey's post-test (**A-E, H**); General Linear Model followed by Tukey's post-test (**I-P**). The behaviors in I-P (n = 12 mice) were assessed 1 day after the last treatment. NE: novel environment, HC: home cage, FLX: fluoxetine, NSFT: novelty-suppressed feeding test, OFT: open-field test, TST: tail suspension test, FST: forced swimming test.

Stress is one of the leading causes of neuropsychiatric disorders, such as anxiety and depression. To explore the mechanisms underlying the anxiolytic and antidepressant effects of fluoxetine, we used CMS exposure as an animal model of anxiety and depression and examined the expression and interaction of nNOS and CAPON in the hippocampal DG of adult mice treated with fluoxetine. Consistent with a previous study, we found that CMS exposure increased the expression of both nNOS and CAPON (Figure 1A), enhanced the association of nNOS with CAPON in the hippocampal DG (Figure 1B), and induced mice to display significant anxiety and depressive behaviors determined using the O-maze test (Figure S1A-B), NSFT (Figure S1C-D), OFT (Figure S1G-H), TST (Figure S1E) and FST (Figure S1F). As expected, administration of fluoxetine (10 mg/kg/d), the most commonly prescribed antidepressant medication, for 28 days, prevented CMS exposure-induced anxiety and depressive behaviors (Figure S1). Furthermore, results from the coimmunoprecipitation experiment demonstrated that chronic fluoxetine treatment attenuated CMS-induced nNOS and CAPON upregulation (Figure 1A) and nNOS- CAPON interaction in the hippocampal DG of mice (Figure 1B). To further determine the effect of fluoxetine on the expression and association of nNOS with CAPON, we also infused CORT (10 μM) into the DG of adult mice using osmotic minipumps (0.25 μL/h) to mimic the behavioral and neurochemical modifications that occur in response to chronic stress. Consistently, chronic CORT infusion produced behavioral modifications similar to those caused by CMS exposure, and chronic fluoxetine treatment prevented the behavioral modifications induced by chronic CORT (Figure S2). Moreover, chronic fluoxetine administration (10 mg/kg/d) attenuated CORT-induced nNOS and CAPON upregulation (Figure 1C) and nNOS-CAPON binding (Figure 1D). Meanwhile, we found that chronic CORT infusion did not affect the integrity of hippocampus by using Nissl staining (Figure S3). Additionally, we incubated cultured neurons with 10 μM CORT alone or in combination with 0.1 μM fluoxetine for 72 hours, and immunoblot experiments showed that fluoxetine significantly abolished the upregulation of nNOS and CAPON expression induced by CORT incubation (Figure 1E). An *in situ* proximity ligation assay (PLA) experiment revealed that fluoxetine reversed the enhancement of PLA signals (nNOS-CAPON coupling) in cultured neurons induced by CORT exposure (Figure 1F-H). Altogether, these results indicated that fluoxetine significantly attenuates chronic stress-induced nNOS and CAPON upregulation and binding.

Previously, we showed that augmenting nNOS-CAPON interaction by introducing AAV-CAPON-L-GFP into the hippocampus resulted in anxiogenic-like behaviors in mice 17. To examine the role of nNOS-CAPON coupling in the behavioral responses to fluoxetine, we infused AAV-CAPON-L-GFP or its control AAV-GFP into the hippocampal DG of mice, and 4 days later, we treated the mice with fluoxetine (10 mg/kg/d) or its vehicle by intraperitoneal administration for 28 days and subsequently exposed these mice to CMS. Consistently, fluoxetine alleviated the anxiogenic and depressive-like phenotypes induced by CMS exposure (Figure 1I-P). Notably, AAV-CAPON-L-GFP effectively infected the hippocampal DG (Figure S4) and prevented the anxiolytic and antidepressant effects of fluoxetine in CMS-exposed mice, as indicated by the reduced time spent in the open arms of the O-maze test (Figure 1I), the prolonged latency to feed in a novel environment in the NSFT (Figure 1K left), the decreased time to enter the inner fields of the OFT (Figure 1M), and prolonged immobility time in the TST and FST (Figure 1O-P), compared to AAV-GFP intrahippocampal DG infusion combined with fluoxetine treatment in CMS-exposed mice. No difference was observed in the number of entries into the arms in the O-maze test (Figure 1J), the latency to feed in the home cage (Figure 1K right), the amount of food consumption (Figure 1L) in the NSFT, or the locomotor activity in the OFT (Figure 1N).

We recently demonstrated that NF-κB signaling mediates the regulation of the nNOS-CAPON association under chronic stress conditions 23. To identify whether NF-κB signaling is involved in the regulation of fluoxetine on the expression and coupling of nNOS and CAPON, we detected levels of p50 and p65, the predominant forms of NF-κB, in the DG of adult mice exposed to CMS and administered fluoxetine for 28 days. During CMS exposure, fluoxetine treatment (10 mg/kg/d i.p.) prevented the CMS-induced increases in p50 and p65 (Figure S5A). These data suggest that fluoxetine prevents CMS-induced NF-κB signaling activation. To further strengthen our findings, we microinfused CORT (10 μM) into the DG of mice and treated the mice with fluoxetine (10 mg/kg/d) for 28 days. The results revealed that CORT markedly increased the levels of p50 and p65 in the DG and that fluoxetine treatment attenuated chronic CORT-induced NF-κB activation (Figure S5B). Furthermore, we incubated cultured neurons with 10 μM CORT alone or in combination with 0.1 μM fluoxetine for 72 h and found that fluoxetine prevented the upregulation of p50 and p65 induced by CORT (Figure S5C). Consistent with these results, there was a noticeable increase in the translocation of p65 to the nucleus in cultured neurons incubated with 10 μM CORT for 72 h, and 0.1 μM fluoxetine attenuated this effect induced by CORT (Figure S5D, E). These results indicated that fluoxetine attenuates chronic stress-induced upregulation of hippocampal NF-κB signaling.

To further determine that fluoxetine regulates nNOS and CAPON expression through NF-κB signaling under chronic stress conditions, we incubated cultured neurons with CORT (10 μM) alone or in combination with fluoxetine (0.1 μM) and NF-κB signaling agonist Betulinic acid (20 μM). Results showed that Betulinic acid significantly reversed the down-regulation of nNOS and CAPON expression induced by fluoxetine (Figure S5F-G).

Collectively, our results suggested that fluoxetine decreases the expression and coupling of nNOS and CAPON in the hippocampal DG under chronic stress conditions via NF-κB signaling.

### Fluoxetine-elevated 5-HT negatively regulated nNOS and CAPON expression and association

**Figure 2:**
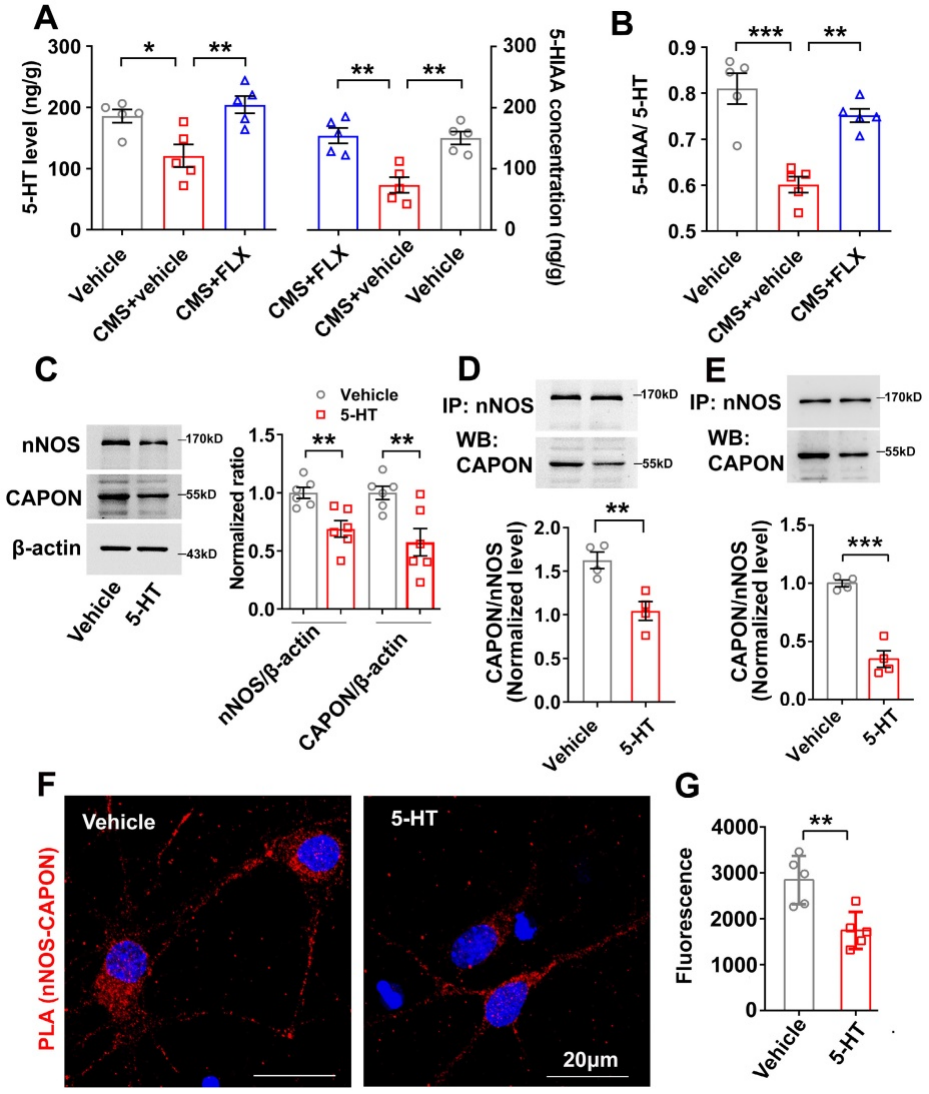
** Fluoxetine-elevated 5-HT negatively regulated nNOS and CAPON expression and association.** (**A-B**) The adult male ICR mice were treated with FLX (10 mg/kg/d) or its vehicle by intraperitoneal administration for 28 consecutive d and exposed to CMS. (**A**) The values of extracellular 5-HT and 5-HIAA contents in hippocampus of mice treated with FLX and exposed to CMS (n = 5, for 5-HT, *F*(2,12) = 8.617; CMS + vehicle versus vehicle: *p = 0.0243; CMS + FLX versus CMS + vehicle: **p = 0.005; for 5-HIAA, *F*(2,12) = 14.33; CMS + vehicle versus vehicle: **p = 0.0019; CMS + FLX versus CMS + vehicle: **p = 0.0013). (**B**) The ratio of 5-HIAA to 5-HT in hippocampus of mice treated with FLX and exposed to CMS (*F*(2,12) = 21.23; CMS + vehicle versus vehicle: ***p = 0.0001; CMS + FLX versus CMS + vehicle: **p = 0.0018). (**C-E**) The adult male ICR mice were treated with 5-HT (10 mg/kg/d) or its vehicle by intraperitoneal administration for 7 consecutive d. (**C**) Representative immunoblots (left) and bar graph (right) showing nNOS and CAPON in the hippocampal DG of mice treated with 5-HT (n = 6, for nNOS, *t*(10)=3.641, 5-HT vs vehicle, **p = 0.0045; for CAPON, *t*(10)=3.231, 5-HT vs vehicle, **p = 0.009). **(D)** 5-HT treatment reduced the amounts of nNOS-CAPON complex in the hippocampal DG of mice (n = 4, *t*(6) = 4.037; 5-HT vs vehicle, **p = 0.0068). (**E**) Representative immunoblots (upper) and bar graph (lower) showing the association of nNOS and CAPON within synaptosomes in hippocampal DG of mice treated with 5-HT (n = 4, *t*(6) = 8.510; 5-HT vs vehicle, ***p = 0.0001). (**F**) Representative confocal images of in situ proximity ligation assay (PLA) between nNOS and CAPON (red) in primary cultured neurons treated with 10 µM 5-HT for 72 hours. Maximum intensity projections of a confocal z-stack including a whole cell were performed to observe the maximum amount of PLA puncta. The neurons were counter-stained with DAPI (blue). Scale bar: 20 µm. **(G)** The quantification of PLA signals (nNOS-CAPON coupling) in primary neurons (n = 5 independent experiments, *t(8)* = 3.699; 5-HT vs vehicle, **p = 0.006). Mean ± SEM, *P < 0.05, **P < 0.01, ***p < 0.001, one-way ANOVA followed by Tukey's post-test (**A-B**); two-tailed Student's t-test (**C-E, G**). FLX: fluoxetine.

Fluoxetine produces its therapeutic effects by elevating extracellular concentrations of 5-HT. We used a liquid fluorescence chromatography detection system to determine the contents of 5-HT and its metabolite, 5-hydroxy-indolacetic acid (5-HIAA), in the hippocampus of mice exposed to CMS and treated with fluoxetine (10 mg/kg/d i.p.) for 28 days. In agreement with other studies, we found that fluoxetine reversed the reduction in 5-HT and 5-HIAA levels caused by CMS exposure (Figure 2A). The turnover ratio of 5-HIAA to 5-HT represents an indicator of cell activity leading to 5-HT release, reuptake and metabolism to 5-HIAA 29. We also found that fluoxetine dramatically prevented the decline in serotonergic activity caused by CMS, as indicated by the ratio of 5-HIAA to 5-HT (Figure 2B).

To study the effect of 5-HT on the expression of nNOS and CAPON, we treated mice with continuous injection of 5-HT (10 mg/kg/d i.p.) for 7 days. The results revealed that 5-HT significantly downregulated the expression of nNOS and CAPON in the hippocampal DG (Figure 2C). Co-IP experiments showed that 5-HT decreased the association of nNOS with CAPON in the DG of adult mice (Figure 2D). More importantly, we compared the association of nNOS with CAPON using synaptosome samples from the DG of vehicle- and 5-HT-treated mice. Our results demonstrated that 5-HT deceased nNOS-CAPON interactions within synaptosomes (Figure 2E). To further explore the effect of 5-HT on nNOS-CAPON coupling, we incubated cultured neurons with 10 μM 5-HT for 72 h, and results from the PLA experiment demonstrated that 5-HT dramatically declined these signals (nNOS-CAPON coupling) in neurons (Figure 2F, G). These results suggested that 5-HT reduces the expression and coupling of nNOS and CAPON in the hippocampal DG.

### nNOS-CAPON coupling was involved in 5-HT1A receptor-mediated behavioral modulation

**Figure 3:**
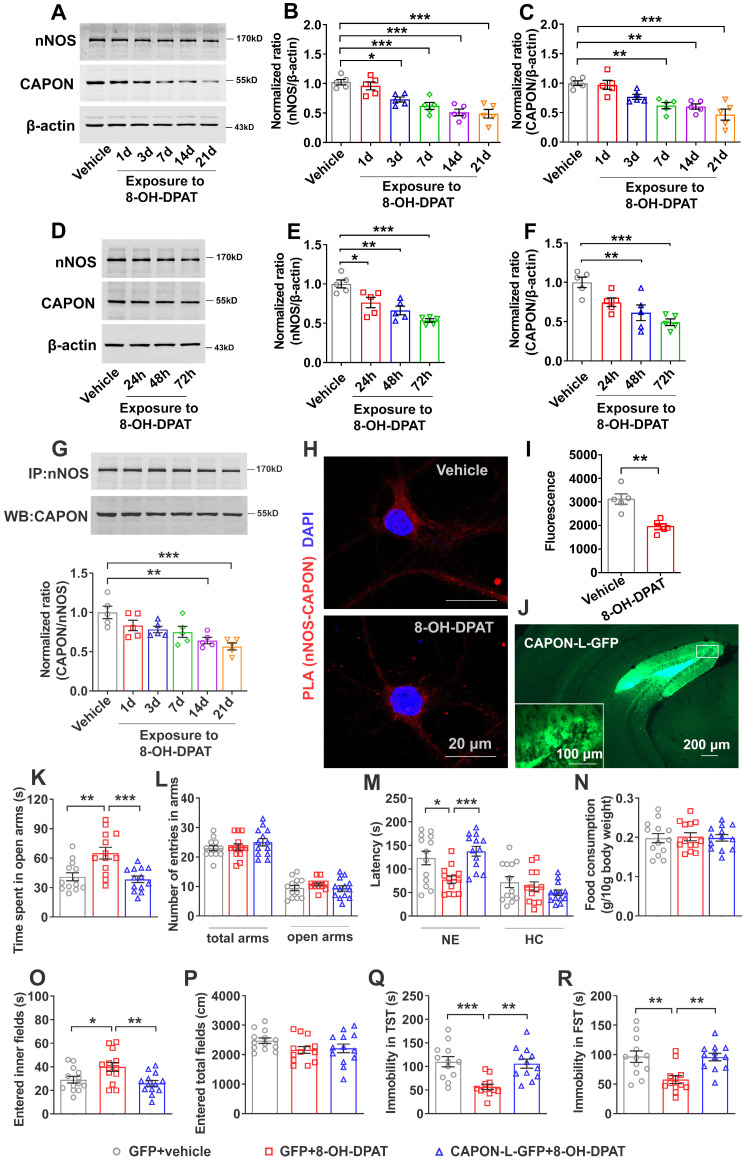
** Augmenting the nNOS-CAPON interaction reversed 5-HT1A receptor agonist-mediated behavioral modulation.** (**A-C**) The adult mice were intraperitoneally treated with 8-OH-DPAT (0.1 mg/kg/d i.p.) for 1, 3, 7, 14 or 21 consecutive days. Representative immunoblots (A) and bar graph (B-C) showing nNOS (n = 5, *F*(5,24) = 15.11, for 1d, *p* =0.9709, for 3d, *p =0.0171, for 7d, ***p =0.0007, for 14d, ***p < 0.0001, for 21d, ***p < 0.0001) and CAPON (n = 5, *F*(5,24) = 12.17, for 1d, *p* =0.9996, for 3d, *p* =0.1202, for 7d, **p =0.0025, for 14d, **p = 0.0016, for 21d, ***p < 0.0001) in the hippocampal DG of mice treated with 8-OH-DPAT. (**D-F**) Representative immunoblots and a bar graph showing nNOS and CAPON levels in the cultured neurons incubated with 10 μM 8-OH-DPAT at 7 DIV for 24, 48, or 72 h (n = 5, for nNOS, *F*(3,16) = 14.85, for 24h, *p = 0.022, for 48h, **p = 0.0013, for 72h, ***p < 0.0001; for CAPON,* F*(3,16) = 9.896, for 24h, *p* = 0.0844, for 48h, **p = 0.0056, for 72h, ***p = 0.0005). (**G**) Representative immunoblots (upper) and bar graph (lower) showing the association of nNOS and CAPON in the hippocampal DG of mice intraperitoneally treated with 8-OH-DPAT (0.1 mg/kg/d i.p.) for 1, 3, 7, 14 or 21 consecutive days (n = 5, *F*(5,24) = 6.604, for 1d,* p* = 0.3711, for 3d, *p* = 0.1332, for 7d, *p* = 0.0637, for 14d, **p = 0.003, for 21d, ***p = 0.0003). (**H**) Representative confocal images of in situ proximity ligation assay (PLA) between nNOS and CAPON (red) in primary cultured neurons treated with 10 µM 8-OH-DPAT for 72 hours. Maximum intensity projections of a confocal z-stack including a whole cell were performed to observe the maximum amount of PLA puncta. The neurons were counter-stained with DAPI (blue). Scale bar: 20 µm. **(I)** The quantification of PLA signals (nNOS-CAPON coupling) in primary neurons (n = 5 independent experiments, *t(8)* = 4.592; 8-OH-DPAT vs vehicle, **p = 0.0018). (**J**) Representative fluorescence image showing the hippocampal DG infected with AAV-CAPON-L-GFP. Scale bar: 200μm. (**K-R**) The adult mice treated with intra-hippocampal microinjection of AAV-CAPON-L-GFP or AAV-GFP in combination with 8-OH-DPAT (0.1 mg/kg/d i.p.) for 28 days. (**K**) The time spent in open arms (*F*(2,36) = 10.32, AAV-GFP + 8-OH-DPAT versus AAV-GFP + vehicle: **p = 0.0019; AAV-CAPON-L-GFP + 8-OH-DPAT versus AAV-GFP + 8-OH-DPAT: ***p = 0.0006) and (**L**) number of entries in the arms (for total arms: *F*(2,36) = 0.9777, *p* = 0.3859; for open arms:* F*(2,36) = 0.8962, *p*= 0.417) in the O-maze test. (**M**) The latency to feed in a novel environment (*W*(2,22.77) = 11.67, AAV-GFP + 8-OH-DPAT versus AAV-GFP + vehicle: *p = 0.0352; AAV-CAPON-L-GFP + 8-OH-DPAT versus AAV-GFP + 8-OH-DPAT: ***p = 0.0004) and in the home cage (*F*(2,36) = 1.458, *p*= 0.2461) and (**N**) food consumption in the home cage (*F*(2,36) = 0.03404, *p*= 0.9666) in the NSFT in adult mice. (**O**) The time of entered inner fields (*F*(2,36) = 5.782, AAV-GFP + 8-OH-DPAT versus AAV-GFP + vehicle: *p = 0.042; AAV-CAPON-L-GFP + 8-OH-DPAT versus AAV-GFP + 8-OH-DPAT: **p = 0.0072) and (**P**) the total distance traveled (*F*(2,36) = 1.928, *p* = 0.1602) in the OFT. (**Q**) The immobility time in the TST (*F*(2,33) = 10.86, AAV-GFP + 8-OH-DPAT versus AAV-GFP + vehicle: ***p = 0.0006; AAV-CAPON-L-GFP + 8-OH-DPAT versus AAV-GFP + 8-OH-DPAT: **p = 0.0014) and (**R**) FST (*F*(2,33) = 8.282, AAV-GFP + 8-OH-DPAT versus AAV-GFP + vehicle: **p = 0.0032; AAV-CAPON-L-GFP + 8-OH-DPAT versus AAV-GFP + 8-OH-DPAT: **p = 0.0039) of the adult mice. The behaviors in J-O (n = 13 mice) and P-Q (n = 12 mice) were assessed 1 day after the last treatment. Mean ± SEM, *P < 0.05, **P < 0.01, ***P < 0.001, one-way ANOVA followed by Tukey's post-test (**A-G, K-L, M-right, N-R**), Welch's ANOVA followed by Dunnett's T3 multiple comparisons test (**M-left**), two-tailed Student's t-test (**I**). NE: novel environment, HC: home cage, NSFT: novelty-suppressed feeding test, OFT: open-field test, TST: tail suspension test, FST: forced swimming test.

Among 5-HT receptors, 5-HT1AR is closely associated with anxiety and depression. To investigate whether 5-HT1AR mediates the effect of 5-HT on the expression and coupling of nNOS and CAPON, we treated mice with the 5-HT1AR agonist 8-OH-DPAT (0.1 mg/kg/d i.p.) for 1, 3, 7, 14 or 21 days. The results revealed that 8-OH-DPAT treatment significantly reduced expression of nNOS in the DG after 3, 7, 14 or 21 days and reduced the expression of CAPON after 7, 14 or 21 days (Figure 3A-C). To further explore the effects of 8-OH-DPAT on the expression of nNOS and CAPON, we incubated cultured hippocampal neurons with 10 μM 8-OH-DPAT for 24, 48, or 72 h. Immunoblotting experiments revealed that expression of nNOS protein was significantly reduced in response to incubation with 8-OH-DPAT for 24, 48 or 72 h, and levels of CAPON were decreased after 48 or 72 h incubation (Figure 3D-F). Additionally, we observed the effect of 8-OH-DPAT on nNOS- CAPON coupling in the hippocampal DG. Coimmunoprecipitation results indicated that 8-OH-DPAT significantly reduced nNOS-CAPON coupling in response to treatment with 8-OH-DPAT for 14 or 21 days (Figure 3G). Results from the PLA experiment showed that incubation with 8-OH-DPAT for 72 h decreased PLA signals (nNOS-CAPON coupling) in cultured neurons (Figure 3H, I). These results suggested that 8-OH-DPAT significantly downregulates the expression and association of nNOS with CAPON in the DG.

To investigate the role of the nNOS-CAPON interaction in 5-HT1AR-mediated anxiety and depressive behaviors, we treated mice with 8-OH-DPAT (0.1 mg/kg/d i.p.) alone or in combination with intrahippocampal DG microinjection of AAV-CAPON-L-GFP for 28 days. Consistently, our data showed that 8-OH-DPAT produced anxiolytic and antidepressant-like effects, while AAV-CAPON-L-GFP effectively infected the hippocampal DG (Figure 3J), abolished the behavioral modification induced by 8-OH-DPAT (Figure 3K-R). These results indicated that treatment with 8-OH-DPAT caused mice to prolong the time spent in the open arms of the zero maze, and transduction of AAV-CAPON-L-GFP in the DG prevented the effects of 8-OH-DPAT (Figure 3K) but did not change the number of entries to the open arms or total arms in the O-maze test (Figure 3L). In the NSFT, 8-OH-DPAT induced a shortened latency to feed in a novel environment, while AAV-CAPON-L-GFP reversed the shortened feeding latency caused by 8-OH-DPAT (Figure 3M, left panel). 8-OH-DPAT either alone or in combination with AAV-CAPON-L-GFP did not affect the latency to feed in home cages (Figure 3M, right panel) or the amount of food consumption within 15 minutes (Figure 3N). In the OFT, AAV-CAPON-L-GFP prevented the increase in the time to enter the central region induced by 8-OH-DPAT treatment (Figure 3O) but did not affect the locomotor activities of mice (Figure 3P). In the TST, hippocampal DG infusion of AAV-CAPON-L-GFP prevented the 8-OH-DPAT-induced reduction in immobility time in mice (Figure 3Q). In the FST, AAV-CAPON-L-GFP significantly prolonged the immobility time of mice caused by 8-OH-DPAT (Figure 3R). These results indicated that intrahippocampal DG infusion of AAV-CAPON-L-GFP abolishes the behavioral modification of 8-OH-DPAT.

**Figure 4:**
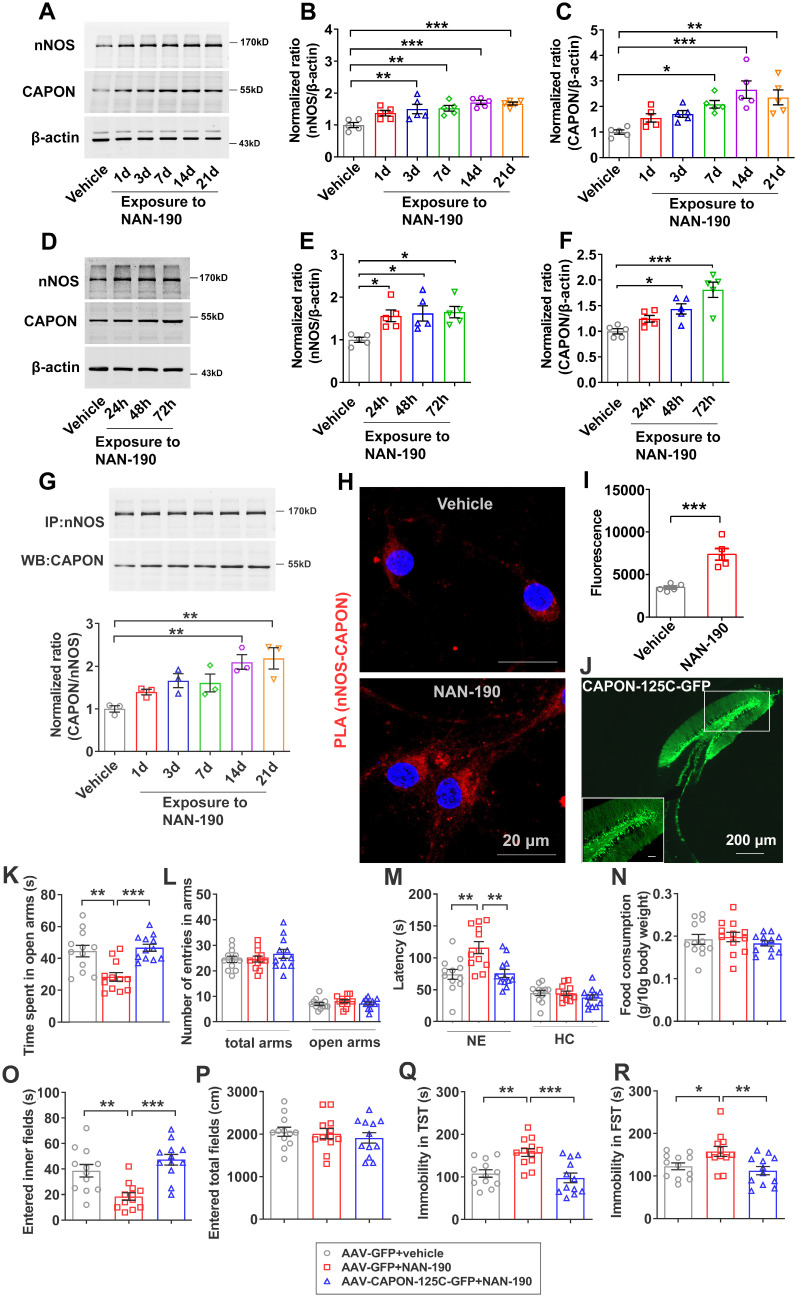
** Disrupting the nNOS-CAPON interaction reversed 5-HT1A receptor antagonist-mediated behavioral modulation.** (**A-C**) The adult mice were intraperitoneally treated with NAN-190 (0.3 mg/kg/d i.p.) for 1, 3, 7, 14 or 21 consecutive days. Representative immunoblots (**A**) and bar graph (**B-C**) showing nNOS (n = 5, *F*(5,24) = 7.962, for 1d, *p* =0.0773, for 3d, **p =0.0084, for 7d, **p =0.0053, for 14d, ***p = 0.0002, for 21d, ***p = 0.0003) and CAPON (n = 5, *F*(5,24) = 7.815, for 1d, *p* =0.4709, for 3d, *p* =0.218, for 7d, *p=0.0166, for 14d, ***p = 0.0002, for 21d, **p = 0.0019) in the hippocampal DG of mice treated with NAN-190. (**D-F**) Representative immunoblots and a bar graph showing nNOS and CAPON levels in the cultured neurons incubated with 10 µM NAN-190 at 7 DIV for 24, 48, or 72 h (n = 5, for nNOS, *F*(3,16) = 5.325, for 24h, *p = 0.0397, for 48h, *p = 0.0217, for 72h, *p = 0.0156; for CAPON, *F*(3,16) = 11.97, for 24h, *p* = 0.328, for 48h, *p = 0.0291, for 72h, ***p = 0.0001). (**G**) Representative immunoblots (upper) and bar graph (lower) showing the association of nNOS and CAPON in the hippocampal DG of mice intraperitoneally treated with NAN-190 (0.3 mg/kg/d i.p.) for 1, 3, 7, 14 or 21 consecutive days (n = 3, *F*(5,12) = 6.929, for 1d, *p* = 0.5742, for 3d, *p* = 0.1259, for 7d, *p* = 0.1789, for 14d, **p = 0.0059, for 21d, **p = 0.0033). (**H**) Representative confocal images of in situ proximity ligation assay (PLA) between nNOS and CAPON (red) in primary cultured neurons treated with 10 µM NAN-190 for 72 hours. Maximum intensity projections of a confocal z-stack including a whole cell were performed to observe the maximum amount of PLA puncta. The neurons were counter-stained with DAPI (blue). Scale bar: 20μm. **(I)** The quantification of PLA signals (nNOS-CAPON coupling) in primary neurons (n = 5 independent experiments, *t(8)* = 5.469; NAN-190 vs vehicle, ***p < 0.001). (**J**) Representative fluorescence image showing the hippocampal DG infected with AAV-CAPON-125C-GFP. Scale bar: 200μm. (**K-R**) The adult mice treated with intra-hippocampal microinjection of AAV-CAPON-125C-GFP or AAV-GFP in combination with NAN-190 (0.3 mg/kg/d i.p.) for 28 days. (**K**) The time spent in open arms (*F*(2,33) = 11.84, AAV-GFP + NAN-190 versus AAV-GFP + vehicle: **p = 0.0011; AAV-CAPON-125C-GFP + NAN-190 versus AAV-GFP + NAN-190: ***p = 0.0003) and (**L**) number of entries in the arms (for total arms: *F*(2,33) = 0.7665, *p*= 0.4728; for open arms:* F*(2,33) = 0.9451, *p* = 0.3989) in the O-maze test. (**M**) The latency to feed in a novel environment (*F*(2,33) = 8.86, AAV-GFP + NAN-190 versus AAV-GFP + vehicle: **p = 0.0022; AAV-CAPON-125C-GFP + NAN-190 versus AAV-GFP + NAN-190: **p = 0.003) and in the home cage (*F*(2,33) = 1.059, *p* = 0.3583) and (**N**) food consumption in the home cage (*F*(2,33) = 0.5872, *p* = 0.5616) in the NSFT in adult mice. (**O**) The time of entered inner fields (*F*(2,33) = 12.92, AAV-GFP + NAN-190 versus AAV-GFP + vehicle: **p = 0.0041; AAV-CAPON-125C-GFP + NAN-190 versus AAV-GFP + NAN-190: ***p < 0.001) and (**P**) the total distance traveled (*F*(2,33) = 0.3936, *p* = 0.6778) in the OFT. (**Q**) The immobility time in the TST (*F*(2,33) = 10.33, AAV-GFP + NAN-190 versus AAV-GFP + vehicle: **p = 0.0037; AAV-CAPON-125C-GFP + NAN-190 versus AAV-GFP + NAN-190: ***p = 0.0005) and (**R**) FST (*F*(2,33) = 5.634, AAV-GFP + NAN-190 versus AAV-GFP + vehicle: *p = 0.0485; AAV-CAPON-125C-GFP + NAN-190 versus AAV-GFP + NAN-190: **p = 0.0082) of the adult mice. The behaviors in (K-R) (n = 12 mice) were assessed 1 day after the last treatment. Mean ± SEM, *P < 0.05, **P < 0.01, ***P < 0.001, one-way ANOVA followed by Tukey's post-test (**A-G, K-R**), two-tailed Student's t-test (**I**). NE: novel environment, HC: home cage NSFT: novelty-suppressed feeding test, OFT: open-field test, TST: tail suspension test, FST: forced swimming test.

To further confirm that 5-HT1AR regulates the expression and coupling of nNOS and CAPON in the hippocampal DG, we treated mice with the 5-HT1AR antagonist NAN-190 (0.3 mg/kg/d i.p.) for 1, 3, 7, 14 or 21 days. Our results showed that expression of nNOS was significantly increased in response to administration of NAN-190 for 3, 7, 14 or 21 days, and expression of CAPON was significantly increased after NAN-190 treatment for 7, 14 or 21 days (Figure 4A-C). Next, we incubated cultured neurons with 10 μM NAN-190 for 24, 48 or 72 h and found that expression of nNOS was significantly increased after 24, 48 or 72 h and that levels of CAPON were increased after incubation for 48 or 72 h (Figure 4D-F). These results indicated that NAN-190 increases the expression of nNOS and CAPON in the hippocampal DG. Most importantly, NAN-190 significantly increased hippocampal DG nNOS-CAPON coupling after treatment for 14 or 21 days (Figure 4G). Meanwhile, incubation with NAN-190 for 72 h in cultured neurons dramatically enhanced PLA signals (nNOS-CAPON coupling) in neurons (Figure 4H, I).

We previously demonstrated that blocking nNOS-CAPON binding using AAV-CAPON-125C-GFP in the hippocampus resulted in anxiolytic-like effects 17. To further confirm that nNOS-CAPON coupling mediates the behavioral modification of 5-HT1AR, we blocked the nNOS-CAPON interaction by infusion of AAV-CAPON-125C-GFP into the DG (Figure 4J) and treated mice with NAN-190 (0.3 mg/kg/d i.p.) for 28 days. As expected, NAN-190-treated mice displayed typical anxiogenic and depressive phenotypes, as indicated by the decreased time spent in the open arms of the O-maze test (Figure 4K), the prolonged latency to feed in the novel environment of the NSFT (Figure 4M, left), the decreased time to enter the inner fields of the OFT (Figure 4O), and prolonged immobility time in the TST and FST (Figure 4Q-R). Intrahippocampal DG microinjection of AAV-CAPON-125C-GFP abolished the effects of NAN-190 on behavioral modifications (Figure 4K-R). Additionally, these treatments had no effect on the number of entries in the open arms and total arms in the O-maze test (Figure 4L), the latency to feed in the home cages (Figure 4M, right), the amount of home cage food consumption (Figure 4N) in the NSFT, or locomotor activity in the OFT (Figure 4P).

Taken together, these results indicated that nNOS-CAPON coupling mediates the behavioral modulation induced by 5-HT1ARs.

### nNOS-CAPON coupling was crucial for 5-HT1AR-modulated neurogenic and synaptogenic effects

**Figure 5:**
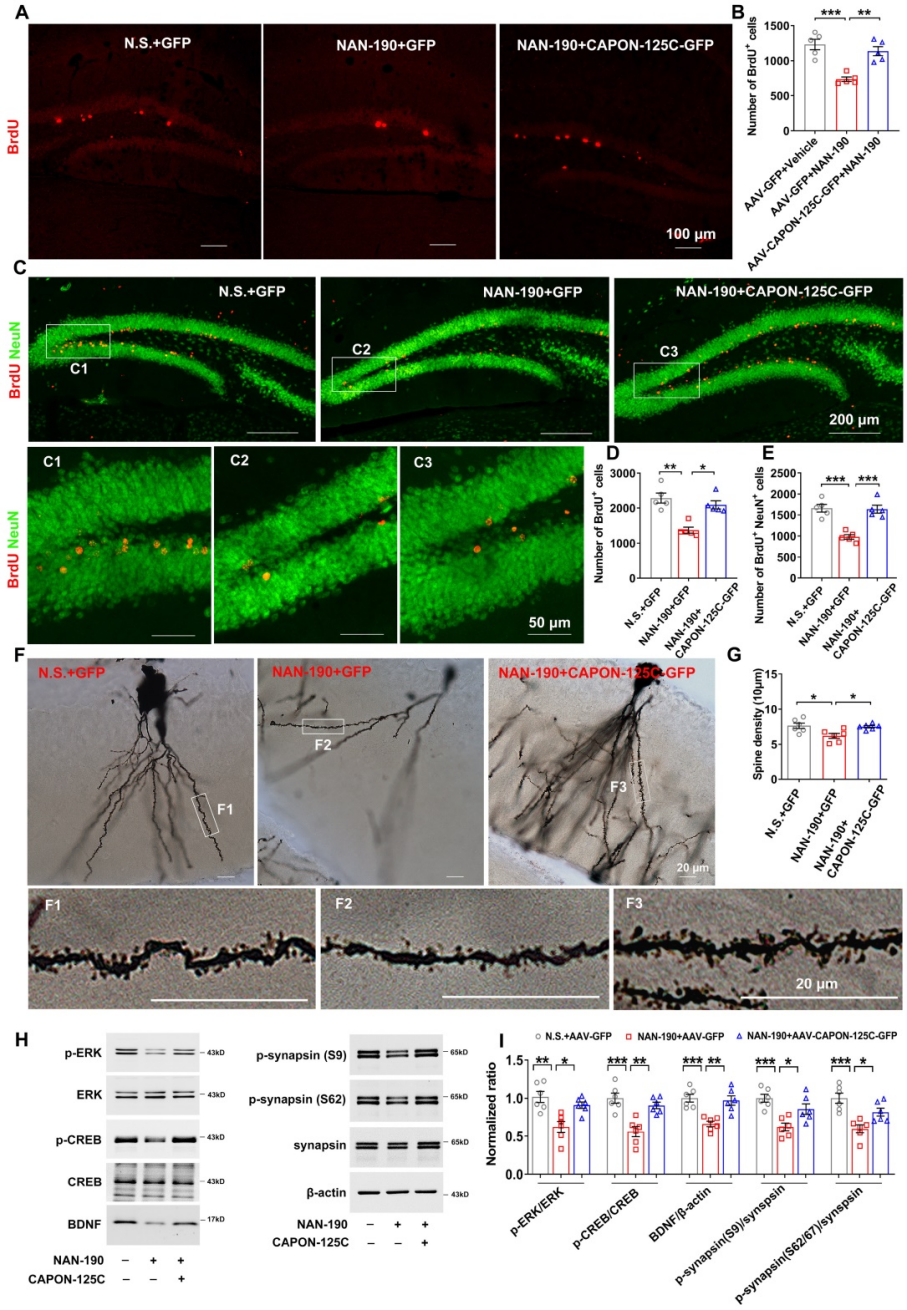
** Dissociation of nNOS-CAPON preventd 5-HT1AR antagonist-induced neurogenic and synaptogenic impairment. (A-I)** The adult mice treated with intra-hippocampal DG microinjection of AAV-CAPON-125C-GFP or AAV-GFP in combination with NAN-190 (0.3 mg/kg/d i.p.) for 28 days. **(A-B)** Representative images **(A)** and bar graph **(B)** showing BrdU^+^ cells (red) in the DG of mice exposed to these treatments at 2 h after BrdU administration (n=5, *F*(2,12) = 19.05, AAV-GFP + NAN-190 versus AAV-GFP + vehicle: ***p = 0.0002; AAV-CAPON-125C-GFP + NAN-190 versus AAV-GFP + NAN-190: **p = 0.0014). Scale bar, 100 µm. **(C-E)** Representative images **(C)** and bar graph showing BrdU^+^ cells **(D)** and BrdU^+^/ NeuU^+^ cells **(E)** in the DG of mice exposed to these treatments at 28 days after BrdU administration (n=5, for BrdU^+^: Kruskal-Wallis statistic = 9.98, AAV-GFP + NAN-190 versus AAV-GFP + vehicle: **p = 0.0047; AAV-CAPON-125C-GFP + NAN-190 versus AAV-GFP + NAN-190: *p = 0.0473; for BrdU^+^/ NeuU^+^: *F*(2,12) = 21.95, AAV-GFP + NAN-190 versus AAV-GFP + vehicle: ***p = 0.0002; AAV-CAPON-125C-GFP + NAN-190 versus AAV-GFP + NAN-190: ***p = 0.0002). BrdU^+^: red, NeuU^+^: green.** (F-G)** Representative images with Golgi-Cox staining **(F)** and bar graph **(G)** showing dendrite spine density of granular cells in the hippocampal DG of mice exposed to different treatments (n = 6, 10 neurons per sample, *F*(2,15) = 6.289, AAV-GFP + NAN-190 versus AAV-GFP + vehicle: *p = 0.0149; AAV-CAPON-125C-GFP + NAN-190 versus AAV-GFP + NAN-190: *p = 0.028). Scale bar, 20 µm. **(H-I)** Representative immunoblots (**H**) and bar graph (**I**) showing p-ERK, p-CREB, p-synapsin, BDNF in the DG of mice with different treatments (n = 6, for p-ERK/ERK: *F*(2,15) = 10.45; AAV-GFP + NAN-190 versus AAV-GFP + vehicle: **p = 0.0014; AAV-CAPON-125C-GFP + NAN-190 versus AAV-GFP + NAN-190: *p = 0.015; for p-CREB: *F*(2,15) = 15.42; AAV-GFP + NAN-190 versus AAV-GFP + vehicle: ***p = 0.0003; AAV-CAPON-125C-GFP + NAN-190 versus AAV-GFP + NAN-190: **p = 0.0024; for BDNF: *F*(2,15) = 13.72; AAV-GFP + NAN-190 versus AAV-GFP + vehicle: ***p = 0.0007; AAV-CAPON-125C-GFP + NAN-190 versus AAV-GFP + NAN-190: **p = 0.0017; for p-syn(S9)/syn: *F*(2,15) = 11.06; AAV-GFP + NAN-190 versus AAV-GFP + vehicle: ***p = 0.0009; AAV-CAPON-125C-GFP + NAN-190 versus AAV-GFP + NAN-190: *p = 0.0278; for p-syn(S62/67)/syn: *F*(2,15) = 12.48; AAV-GFP + NAN-190 versus AAV-GFP + vehicle: ***p = 0.0004; AAV-CAPON-125C-GFP + NAN-190 versus AAV-GFP + NAN-190: *p = 0.0395). Mean ± SEM, *P < 0.05, **P < 0.01, ***P < 0.001, one-way ANOVA.

Hippocampal neurogenesis is implicated in the regulation of 5-HT1AR-mediated anxiety and depressive behaviors as well as the response to chronic SSRIs. To investigate whether blocking nNOS-CAPON interaction affects 5-HT1AR-mediated neuronal proliferation, we delivered AAV-CAPON-125C-GFP or AAV-GFP into the bilateral hippocampal DG of adult mice, and 4 days later, we treated these mice with NAN-190 (0.3 mg/kg/d i.p.) or vehicle for 28 days. Twenty-four hours after the last NAN-190 injection, mice were injected with BrdU (100 mg/kg, i.p.) and sacrificed 2 h later. We found that NAN-190 significantly decreased the number of BrdU-positive cells in the granule cell layer, while dissociation of nNOS with CAPON by AAV-CAPON-125C-GFP reversed the effects of NAN-190 on cell proliferation (Figure 5A-B). Meanwhile, to further confirm the role of nNOS-CAPON interaction in 5-HT1AR-mediated neuronal proliferation, we infused AAV-CAPON-L-GFP or AAV-GFP into the hippocampal DG of mice, and 4 days later, we treated mice with 8-OH-DPAT (0.1 mg/kg/d i.p.) or vehicle for 28 days. The results showed that 8-OH-DPAT increased the number of BrdU-positive cells, while augmenting nNOS-CAPON interaction by AAV-CAPON-L-GFP reversed the effects of 8-OH-DPAT on cell proliferation (Figure S6A-B).

To test the role of nNOS-CAPON interaction in 5-HT1AR-mediated neuronal differentiation of adult newborn cells, we delivered AAV-CAPON-125C-GFP or AAV-GFP into the bilateral DG of adult mice, and 4 days later, we treated these mice with NAN-190 (0.3 mg/kg/d i.p) or vehicle for 28 days. At 4 days after AAV-CAPON-125C-GFP or AAV-GFP infusion, mice were injected with BrdU (50 mg/kg, i.p., 4 times at 12 h intervals) and then sacrificed 28 days after the last BrdU injection. The results demonstrated that NAN-190 decreased the number of BrdU^+^ (Figure 5C-D) and BrdU^+^/NeuN^+^ cells (Figure 5C, E) in the DG, and AAV-CAPON-125C-GFP rescued the effects of NAN-190 on neuron production (Figure 5C-E). In contrast, we delivered AAV-CAPON-L-GFP or AAV-GFP into the hippocampal DG of mice, and 4 days later, treated mice with 8-OH-DPAT (0.1 mg/kg/d i.p.) or vehicle for 28 days. The results showed that 8-OH-DPAT increased the number of BrdU^+^ (Figure S6C-D) and BrdU^+^/NeuN^+^ cells (Figure S6C, E), and AAV-CAPON-L-GFP reversed the effects of 8-OH-DPAT on neuron production (Figure S6C-E).

Deficits in 5-HT-modulated synaptic plasticity impact the action of SSRIs. To determine whether blocking nNOS-CAPON binding is crucial for 5-HT1AR-mediated synaptogenesis, we infused AAV-CAPON-125C-GFP or AAV-GFP into the hippocampal DG of mice, treated the mice with NAN-190 or vehicle for 28 days, and measured dendritic spine density of granule cells. NAN-190, antagonist of 5-HT1AR decreased the dendritic spine density of granule cells, and AAV-CAPON-125C-GFP rescued the effect of NAN-190 (Figure 5F-G). To further confirm our findings, we infused AAV-CAPON-L-GFP or AAV-GFP into the hippocampal DG of mice, and 4 days later, we treated mice with 8-OH-DPAT (0.1 mg/kg/d i.p.) or vehicle for 28 days. The results demonstrated that AAV-CAPON-L-GFP blocked 8-OH-DPAT-induced increases in dendritic spine density of granule cells (Figure S6F-G).

To investigate the molecular mechanism underlying the hippocampal neuroplasticity changes in the postsynaptic 5-HT1AR-linked nNOS-CAPON interaction, we examined levels of phosphorylated ERK, CREB and synapsin and expression of BDNF, important signaling molecules related to neuroplasticity. AAV-CAPON-125C-GFP ameliorated the NAN-190-induced reduction in p-ERK, p-CREB, BDNF and p-synapsin in the DG of mice (Figure 5H-I), whereas AAV-CAPON-L-GFP abolished the 8-OH-DPAT-triggered p-ERK, p-CREB, BDNF and p-synapsin upregulation (Figure S6H-I).

In conclusion, these findings demonstrated that the nNOS-CAPON interaction is crucial for 5-HT1AR-mediated neurogenic and synaptogenic effects.

## Discussion

**Figure 6:**
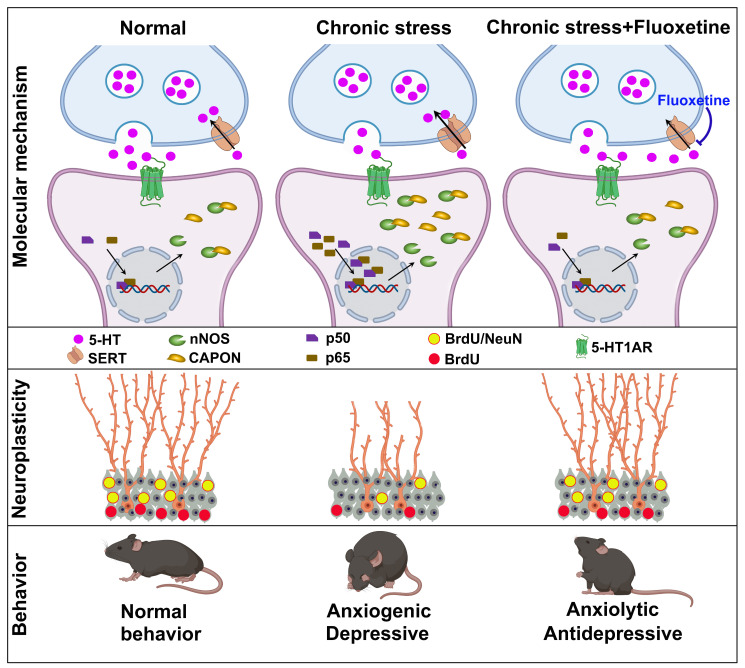
**A model of the signaling cascade whereby fluoxetine regulates anxiety and depressive behaviors.** Chronic fluoxetine treatment elevates 5-HT levels in the hippocampus, activates 5-HT1A receptor via promoting 5-HT neurotransmission, decreases p50/p65 entry into the nucleus, reduces nNOS-CAPON expression and coupling, and enhances hippocampal DG neurogenesis and neuronal differentiation, thereby modulating anxiety and depressive behaviors.

SSRIs have been used for treating various psychiatric disorders, especially depression and anxiety 30. Although the mechanisms underlying the behavioral effects of SSRIs have been extensively studied, the molecular mechanism by which fluoxetine ameliorates symptoms remains mostly unknown. In the present study, we demonstrated that chronic treatment with the SSRI fluoxetine prevented chronic stress-induced nNOS-CAPON binding. Promoting the nNOS-CAPON association in the hippocampal DG weakened the anxiolytic and antidepressant effects of fluoxetine in a CMS animal model. Meanwhile, the increase in serotonin levels induced by fluoxetine activates 5-HT1ARs and enhances hippocampal neurogenesis and dendritic spine remodeling, regulating anxiety and depressive behaviors by affecting the nNOS-CAPON interaction. Therefore, we revealed new molecular substrates for the behavioral and neuroplasticity responses to fluoxetine (Figure 6).

Clinical studies evidenced that CAPON is associated with the pathological anxiety, included the posttraumatic stress disorder (PTSD) and obsessive-compulsive disorder (OCD) 31-33. Our previous studies have also consistently suggested the involvement of CAPON in anxiety modulation 17, 23. However, Freudenberg et al. found that CAPON overexpression in dorsal hippocampus had no effects on anxiety related behaviors 34, which may be due to the differential contribution of hippocampus along the dorsoventral axis to the anxiety regulation 35, 36. Therefore, present study was performed to explore the potential clinical significance of hippocampal DG nNOS-CAPON coupling in treatment of neuropsychiatric disorders.

5-HT is an ancient, evolutionarily conserved neurotransmitter for emotional and cognitive processing that is synthesized and released by 5-HTergic neurons, which are primarily located in the raphe nuclei 6. SSRIs inhibit 5-HT reuptake into raphe nuclei neurons, while chronic treatment leads to increased extracellular 5-HT levels in multiple brain regions innervated by the terminals of 5-HTergic neurons, including the ventral hippocampus 10. Consistently, we found that CMS decreased extracellular 5-HT concentrations and serotonergic ratios (5-HIAA to 5-HT) in the hippocampus, while chronic fluoxetine treatment rescued the downregulation of 5-HT and the ratio of 5-HIAA to 5-HT. More importantly, 5-HT decreased the association between nNOS and CAPON in the hippocampal DG and cultured neurons, indicating a crucial role of chronic fluoxetine treatment in elevating 5-HT to effect on the nNOS-CAPON association. There is extensive research evidence a vital role of 5-HT neurotransmission in the therapeutic effects of SSRIs 6, 37. Interestingly, 5-HT fibers usually lack direct synaptic contacts. Moreover, in many cases, 5-HT receptors expressed in neurons that do not receive direct serotonergic innervation 7, 38, indicating that 5-HT is released diffusely in the hippocampus, like in other areas of the brain, and function as a neuromodulator in the brain. Increasing research attention has been paid to serotonergic targets other than SERT, such as distinct 5-HT receptors, in the development of therapeutic drugs for anxiety and depression.

There are at least fourteen distinct 5-HT receptors, and almost all identified 5-HT receptor subtypes are expressed in rodent hippocampal circuits 6, 7. Reportedly, 5-HT1AR has the highest affinity for 5-HT among all 5-HT receptor subtypes 7. Activation of somatodendritic 5-HT1A autoreceptors in the raphe nuclei by 5-HT or 5-HT1A agonists induces hyperpolarization, and reduces 5-HT synthesis, turnover and release at the axon terminals in projection areas 6, 39, 40. Unlike autoreceptors, activation of postsynaptic 5-HT1A heteroreceptors results in hyperpolarizing response on pyramidal neurons of forebrain regions such as the prefrontal cortex, hippocampus, and amygdala 41, 42. Here, we focused on the 5-HT1A receptor, as numerous studies suggested that 5-HT1A heteroreceptors were implicated in the response to antidepressant 9. Herein, we found that systemic administration of 8-OH-DPAT, a selective 5-HT1AR agonist, decreased the expression and association of nNOS and CAPON in the hippocampal DG, while NAN-190, a selective 5-HT1AR antagonist, augmented nNOS and CAPON expression and coupling. Meanwhile, experimental results showed that CAPON expression changed later than nNOS in response to drug treatments, implying that nNOS may be an upstream signal of CAPON expression. Indeed, we previously reported that nNOS-derived NO accounts for CMS-induced CAPON expression 17. Moreover, 5-HT1AR modulates nNOS-CAPON binding in the hippocampal DG, likely because its agonist and antagonist affect nNOS and CAPON expression.

Multiple evidences illuminated that CMS-induced glucocorticoids overproduction is contributed to stress-related depressive and anxiety-like behaviors 22, 23, thus, chronic corticosterone administration and CMS were performed to model anxiety and depression states, and current results showed that fluoxetine ameliorates CMS/CORT induced nNOS and CAPON expression and coupling. Although our previous studies demonstrated that CAPON overexpression promoted nNOS-CAPON coupling, but not CAPON overexpression, regulates anxiety behavior, since anxiogenic effect induced by hippocampal CAPON overexpression was disappeared in nNOS-knockout mice 17. Here, we cannot completely rule out whether the amelioration of chronic stress-induced behavioral effects by fluoxetine is mediated by the changes of CAPON expression. Another question we are interested in is what are the molecular mechanisms by which fluoxetine regulates nNOS and CAPON expression under chronic stress conditions. We found that fluoxetine reverses chronic stress-activated NF-κB signaling, and NF-κB agonists Betulinic acid significantly alleviated the down-regulation of nNOS and CAPON expression induced by fluoxetine under chronic stress conditions. These findings are consistent with our previous study showing that NF-κB modulates the expression of nNOS and CAPON in the hippocampus 23.

Interestingly, we determined that nNOS-CAPON coupling in the hippocampal DG mediates the behavioral modifications of 5-HT1ARs. However, we cannot rule out whether postsynaptic 5-HT1A receptors in other brain regions receiving serotonergic innervation are associated with the regulation of anxiety and depressive behaviors by fluoxetine via affecting nNOS-CAPON coupling. Furthermore, there is increasing evidence that autoreceptor and heteroreceptor of the 5-HT1A function opposite in the regulation of anxiety and depressive-like behaviors. Therefore, future attempts should focus on specifically modulating the auto- or hetero-5-HT1ARs to elucidate the role of the nNOS-CAPON interaction in the behavioral modification of fluoxetine-elevated 5-HT. Additionally, our results demonstrated that levels of the nNOS-CAPON complex were altered after treatment with the 5-HT1AR agonist or antagonist for 14 days, which occurred later than 5-HT treatment for 7 days. These results indicate the possibility that contributions from other 5-HT receptors cannot be ruled out. For example, the role of 5-HT2C receptor (5-HT2CR) in the regulation of anxiety has been widely recognized 43, 44. Thus, other 5-HT receptors, particularly 5-HT2CR would be further studied in our future work.

The contradiction between the rapid increase in serotonin levels and the delayed onset of actions leads us to postulate that structural or functional changes may be involved in the therapeutic effect of SSRIs. Results from a series of studies have shown that hippocampal neurogenesis and synaptic plasticity play critical roles in the antidepressant effects of fluoxetine, and DG 5-HT1A heteroreceptors are required for the neurogenic effects of chronic fluoxetine treatment 8, 11. There are studies also have shown that CAPON and CAPON-mediated protein-protein interaction are involved in the regulation of dendritic spine plasticity 31, 45, 46. Consistently, our present study found that dissociation of nNOS with CAPON in the DG by AAV-CAPON-125C-GFP rescued the effects of NAN-190 on cell proliferation and neuron production. We also demonstrated that AAV-CAPON-L-GFP counteracted the 8-OH-DPAT-induced upregulation of spine density in hippocampal DG granule cells and abolished the anxiolytic effect of 8-OH-DPAT, whereas AAV-CAPON-125C-GFP rescued the NAN-190-induced downregulation of spine density and reversed the anxiogenic effect of NAN-190. Collectively, these lines of evidence indicated that increasing 5-HT levels induced by chronic fluoxetine treatment partially facilitate 5-HT neurotransmission via 5-HT1A receptors, dissociate nNOS from CAPON in the DG, and promote hippocampal neural plasticity, thereby modulating anxiety and depressive behaviors. In the hippocampus, 5-HT1A heteroreceptors are primarily located on nonserotonergic cells, including pyramidal neurons, dentate gyrus granule cells, and GABAergic interneurons 7, 11. Given that the expression of 5-HT1AR exhibits a unique pattern with the most prominent being in the DG ventral subregion, and the ventral hippocampus is mainly involved in regulating stress and emotional behaviors 12. Future work should examine the suppression of inhibitory neuronal vs. granular 5-HT1A receptors in the ventral DG, potentially elucidating their distinct regulation of nNOS-CAPON coupling on neuroplasticity and the behavioral effects of fluoxetine.

In the past decade, the molecular mechanisms underlying the clinical improvement associated with fluoxetine have been thoroughly investigated. However, the underlying molecular mechanisms of postsynaptic 5-HT1A receptor stimulation by fluoxetine on behavior and neural plasticity in the hippocampal DG remain to be fully elucidated. 5-HT1AR couples primarily to G-alpha(i3) in raphe area 5-HT neurons and G-alpha(o) in hippocampal neurons, which may be the substrates of the differential signaling in these cells 47. Hippocampal 5-HT1A receptor signaling via CAMKII and ERK1/2 to CREB activity increases BDNF expression 48, 49. It is generally believed that ERK-CREB-BDNF signaling mediates neural plasticity, leading to reduced anxiety and depressive-like behaviors 18, 50-52. Our data demonstrated that AAV-CAPON-L-GFP neutralized the 5-HT1AR agonist 8-OH-DPAT-stimulated phosphorylation of ERK and CREB and BDNF expression, whereas AAV-CAPON-125C-GFP rescued the 5-HT1AR antagonist NAN-190-induced downregulation of p-ERK, p-CREB, and BDNF levels. It is well accepted that the effects of BDNF on synaptic transmission are initiated upon binding of BDNF to its receptor tyrosine kinase, TrkB, and subsequently activates various downstream signaling pathways 53. CAPON interacts with synapsin I through its PTB domain, and deletion of synapsin I alters the subcellular localization of nNOS and CAPON, and impairs dendritic arborization 54. Moreover, a causal link of BDNF-TrkB and ERK mediated synapsin phosphorylation with facilitation of neurotransmitter glutamate release was shown to enhance granule cells dendritic development and maturation 55, 56. In this study, augmenting nNOS- CAPON binding neutralized 8-OH-DPAT-induced synapsin phosphorylation at P-Sites 4/5, corresponding to serine 62/67, and P-site 1, corresponding to serine 9, whereas dissociation of nNOS with CAPON rescued the NAN-190-induced downregulation of p-synapsin. Therefore, 5-HT transmission-induced nNOS-CAPON dissociation may be an initiator of synapsin I phosphorylation, leading to glutamate release and enhancement of synaptic transmission, which needs to be further examined in future research.

In conclusion, our data demonstrate the important role of nNOS-CAPON uncoupling in the anxiolytic and antidepressant responses to fluoxetine, identifying novel molecular substrates for the responses to the classical SSRI fluoxetine. Given that nNOS-CAPON coupling mediates anxiety behaviors, posttraumatic stress disorder and ischemia-induced impairment of structural plasticity 17, 57, 58, our present study strengthens the observation that nNOS-CAPON coupling, an improved intervention target other than SERT or 5-HT receptors, represents a target for treating neuropsychiatric disorders.

## Supplementary Material

Supplementary figures.

## Abbreviations

SSRI: selective serotonin reuptake inhibitor
SERT: serotonin reuptake transporters
5-HT: 5-hydroxtryptamine (serotonin)
5HT1AR: 5-HT1A receptor
DG: dentate gyrus
NSCs: neural stem cells
nNOS: neuronal nitric oxide synthase
CAPON: carboxy-terminal PDZ ligand of nNOS (also known as NOS1AP)
ERK: extracellular signal regulated kinase
CREB: cAMP-response element binding protein
BDNF: brain-derived neurotrophic factor
CORT: corticosterone
CMS: chronic mild stress
OFT: open-field test
NSFT: novelty-suppressed feeding test
O-maze: elevated zero maze
FST: forced swimming test
TST: tail suspension test
EtOH: ethyl alcohol
CO-IP: coimmunoprecipitation
PLA: Duolink proximity ligation assay
RRIDs: Research Resource Identifiers
DIV: days *in vitro*
ANOVA: Analysis of Variance
