# Supplementary material for: Requirement of hippocampal DG nNOS-CAPON dissociation for the anxiolytic and antidepressant effects of fluoxetine
Source: Theranostics. 2022 May 1;12(8):3656–75. doi: 10.7150/thno.70370 (PMC9131266; doi:10.7150/thno.70370)
Supplement: Supplementary file 1 — Supplementary figures. [file thnov12p3656s1.pdf]

Supplementary Information

Requirement of hippocampal DG nNOS-CAPON dissociation for the anxiolytic and antidepressant effects of fluoxetine

Hu-Jiang Shi[1#], Dan-Lian Wu[2#], Rong Chen[1#], Na Li[1], Li-Juan Zhu[1, 3*]

Correspondence to: (zhulj@seu.edu.cn).

This PDF file includes:

Supplementary Figures 1 to 6

Supplementary Figures

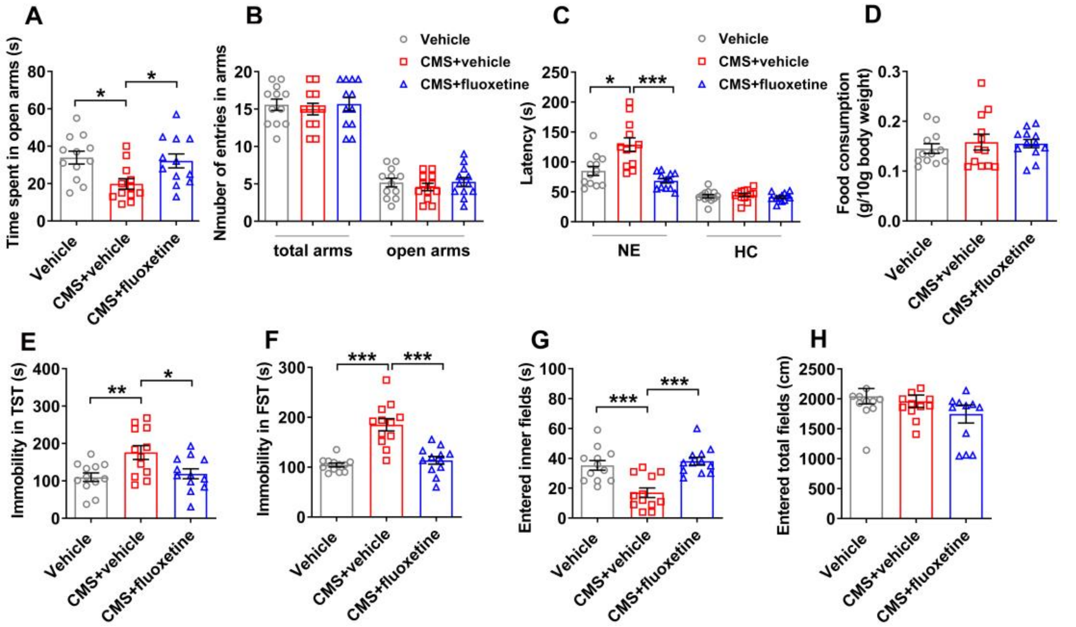

Figure S1 Fluoxetine reversed chronic stress-induced behavioral modification. The adult male ICR mice were treated with fluoxetine (10 mg/kg/d) or its vehicle by intraperitoneal administration for 28 consecutive d and exposed to CMS. (**A**) The time spent in open arms ($F(2,33) = 5.233$, CMS + vehicle versus vehicle: *$p = 0.0149$; CMS + fluoxetine versus CMS + vehicle: *$p = 0.0360$) and (**B**) number of entries in the arms (for total arms: $F(2,33) = 0.1982$, $p = 0.8212$; for open arms: $F(2,33) = 0.4254$, $p = 0.6571$) in the O-maze test. (**C**) The latency to feed in a novel environment ($W(2,18.73) = 13.14$, CMS + vehicle versus vehicle: *$p = 0.0126$; CMS + fluoxetine versus CMS + vehicle: ***$p = 0.0005$) and in the home cage ($F(2,33) = 0.4669$, $p = 0.6310$) and (**D**) food consumption in the home cage ($F(2,33) = 0.3342$, $p = 0.7183$) in the novelty-suppressed feeding test in adult mice. (**E**) The immobility time in the TST ($F(2,33) = 5.925$, CMS + vehicle versus vehicle: **$p = 0.0088$; CMS

+ fluoxetine versus CMS + vehicle: **p* = 0.0262) and (**F**) FST (*W*(2,18.35) = 18.45, CMS + vehicle versus vehicle: ****p* < 0.0001; CMS + fluoxetine versus CMS + vehicle: ****p* = 0.0004) of the adult mice. (**G**) The time of entered inner fields (*F*(2,33) = 14.42, CMS + vehicle versus vehicle: ****p* = 0.0004; CMS + fluoxetine versus CMS + vehicle: ****p* < 0.0001) and (**H**) the total distance traveled (*F*(2,33) = 1.418, *p* = 0.2566) in the OF test. The behaviors in (**A-H**) (n = 12 mice) were assessed 1 day after the last treatment. Data were normally distributed with Shapiro-Wilk test and one-way ANOVA followed by Tukey's multiple comparisons test (**A-B**, **C**-right, **D-E**, **G-H**), or Welch ANOVA with Dunnett's T3 multiple comparisons test (**C**-left and **F**) were carried out to test significance, **P* < 0.05, ***P* < 0.01, ****P* < 0.001. Graphs show Mean ± SEM. NE: novel environment, HC: home cage.

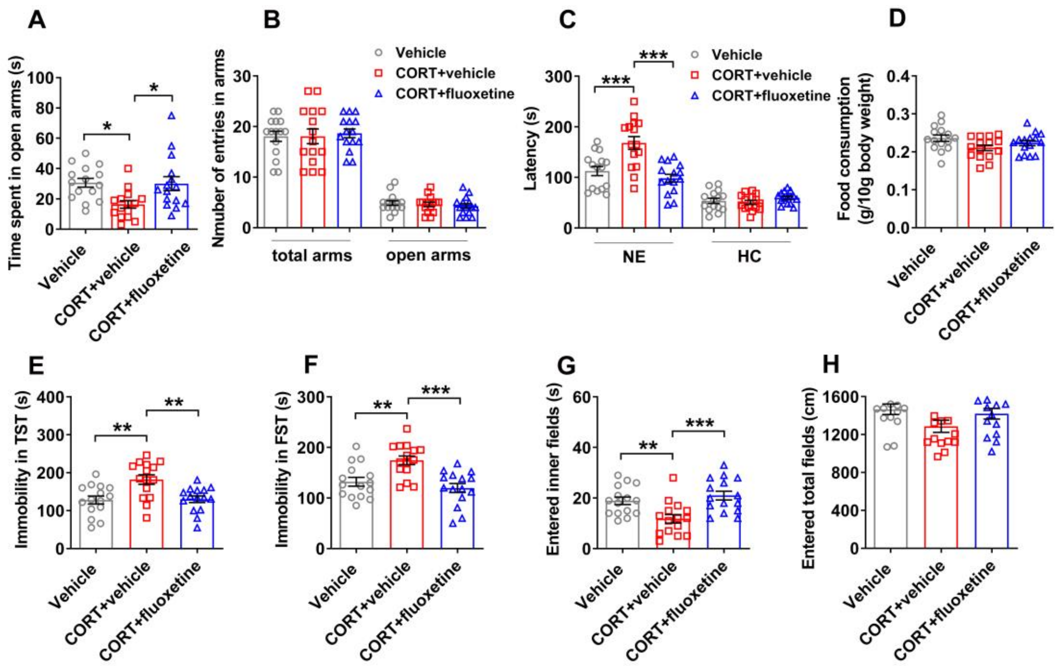

Figure S2 Fluoxetine reversed chronic CORT-induced behavioral modification. The adult male ICR mice were treated with CORT (10 μM) alone or in combination

with fluoxetine (10 mg/kg/d) or its vehicle by intraperitoneal administration for 28 consecutive d. **(A)** The time spent in open arms (F(2,42) = 5.594, CORT + vehicle versus vehicle: *p = 0.014; CORT + fluoxetine versus CORT + vehicle: *p = 0.0186) and **(B)** number of entries in the arms (for total arms: F(2,42) = 0.09206, p = 0.9122; for open arms: F(2,42) = 0.4439, p = 0.6445) in the O-maze test. **(C)** The latency to feed in a novel environment (F(2,42) = 13.47, CORT + vehicle versus vehicle: ***p = 0.001; CORT + fluoxetine versus CORT + vehicle: ***p < 0.0001) and in the home cage (F(2,42) = 1.273, p = 0.2906) and **(D)** food consumption in the home cage (F(2,42) = 2.859, p = 0.0686) in the novelty-suppressed feeding test in adult mice. **(E)** The immobility time in the TST (F(2,42) = 8.214, CORT + vehicle versus vehicle: **p = 0.0025; CORT + fluoxetine versus CORT + vehicle: **p = 0.0038) and **(F)** FST (F(2,42) = 10.9, CORT + vehicle versus vehicle: **p = 0.0036; CORT + fluoxetine versus CORT + vehicle: ***p = 0.0002) of the adult mice. **(G)** The time of entered inner fields (F(2,42) = 8.741, CORT + vehicle versus vehicle: **p = 0.0097; CORT + fluoxetine versus CORT + vehicle: ***p = 0.0008) and **(H)** the total distance traveled (F(2,42) = 2.54, p = 0.0909) in the OF test. The behaviors in **(A-H)** (n = 15 mice) were assessed 1 day after the last treatment. Data were normally distributed with Shapiro-Wilk test and one-way ANOVA followed by Tukey's multiple comparisons test **(A-H)**, was carried out to test significance, *P < 0.05, **P < 0.01, ***P < 0.001, ****P < 0.0001. Graphs show Mean ± SEM. NE: novel environment, HC: home cage.

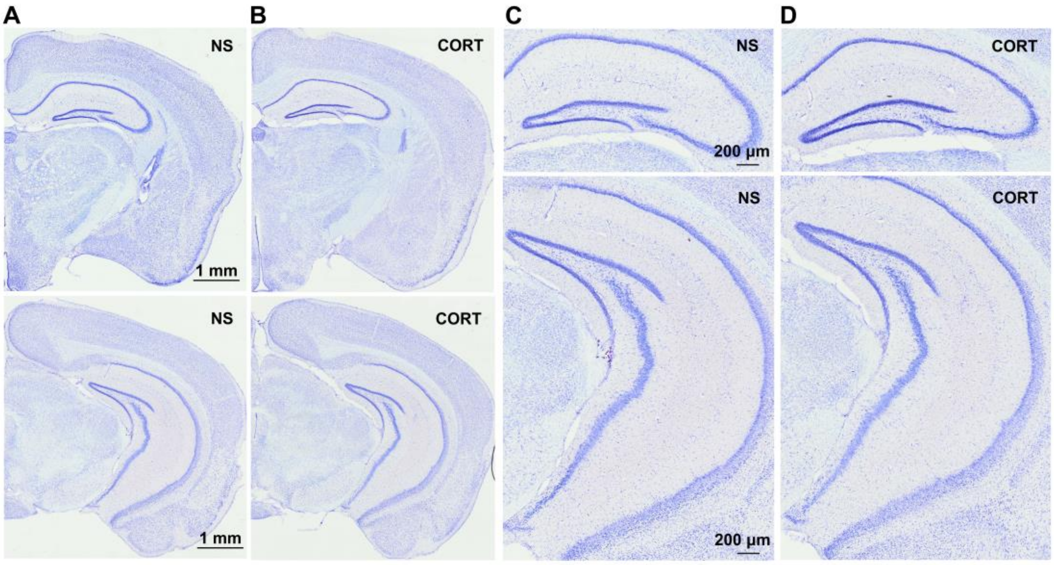

Figure S3 Chronic CORT infusion did not alter the integrity of the infused hippocampus. Corticosterone (10 μM) was delivered into the DG of the hippocampus by microinjection for 28 consecutive d. (A-D) Representative images showing chronic CORT infusion did not alter the integrity of the infused hippocampus by using Nissl staining. Scale bar: 1mm (A-B), 200μm (C-D).

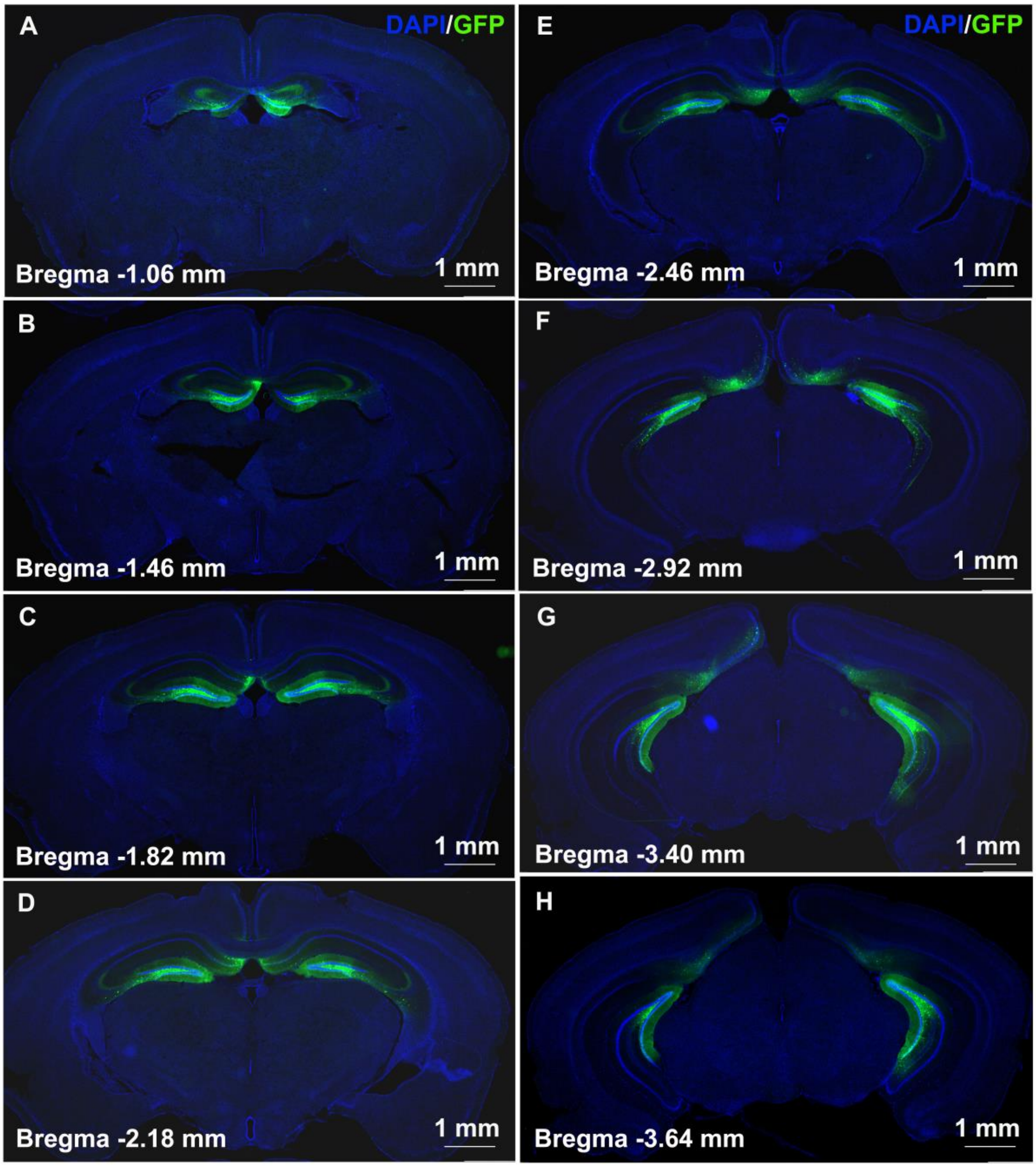

Figure S4 The expression of virus across the anterior posterior of hippocampus. **(A-H)** The AAV-CAPON-L-GFP was injected into the DG of the hippocampus, and effectively infected the hippocampus, produced considerable CAPON-L-GFP. Scale bar: 1mm.

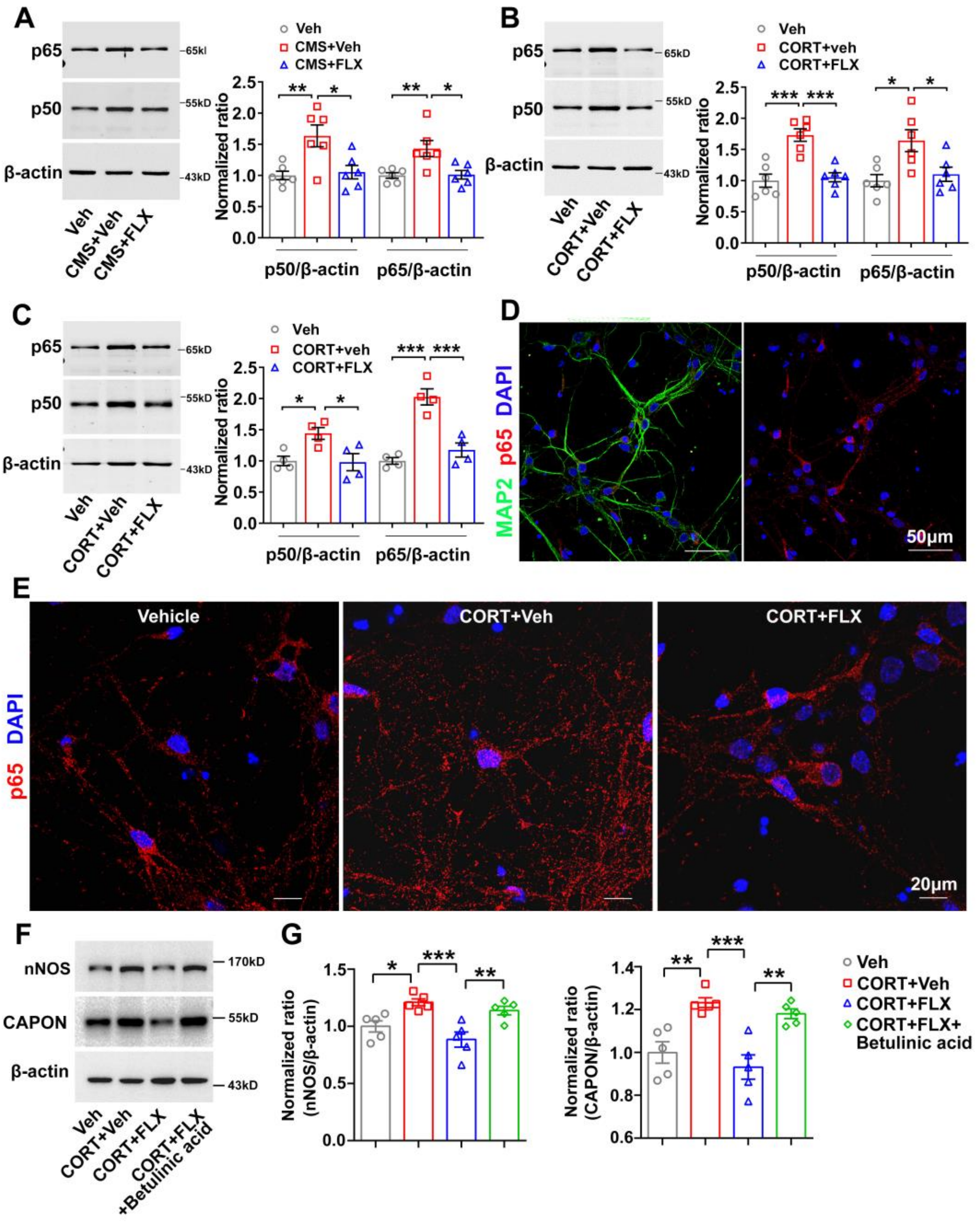

Figure S5 Fluoxetine prevented chronic stress-induced NF-κB signaling activation. (A) The adult male mice were treated with fluoxetine (10 mg/kg/d) or its vehicle by intraperitoneal administration for 28 consecutive d and exposed to CMS. Representative immunoblots (left) and bar graph (right) showing hippocampal DG p50 ($F(2,15) = 7.987$, CMS + vehicle versus vehicle: $^{**}p = 0.0069$; CMS + FLX versus CMS + vehicle: $^{*}p = 0.0127$) and p65 ($F(2,15) = 7.82$, CMS + vehicle versus vehicle: $^{**}p = 0.009$; CMS + FLX versus CMS + vehicle: $^{*}p = 0.0109$) levels in adult

male ICR mice ($n = 6$). **(B)** The adult male mice were treated with CORT (10 μM) alone or in combination with fluoxetine (10 mg/kg/d) or its vehicle by intraperitoneal administration for 28 consecutive d. Representative immunoblots (left) and bar graph (right) showing p50 ($F(2,15) = 18.95$, CORT + vehicle versus vehicle: ***$p = 0.0002$; CORT + FLX versus CORT + vehicle: ***$p = 0.0004$) and p65 ($F(2,15) = 6.702$, CORT + vehicle versus vehicle: *$p = 0.0102$; CORT + FLX versus CORT + vehicle: *$p = 0.0302$) levels in hippocampal DG ($n = 6$). **(C)** Representative immunoblots (left) and bar graph (right) showing p50 ($F(2,9) = 6.072$, CORT + vehicle versus vehicle: *$p = 0.0392$; CORT + FLX versus CORT + vehicle: *$p = 0.0318$) and p65 ($F(2,9) = 28.03$, CORT + vehicle versus vehicle: ***$p = 0.0002$; CORT + FLX versus CORT + vehicle: ***$p = 0.0007$) levels in the cultured neurons incubated with 10 μM CORT alone or in combination with 0.1μM fluoxetine or vehicle for 72 h ($n = 4$). **(D)** Representative p65 immunofluorescence in cultured neurons (red, p65; green, MAP-2; blue, DAPI). DAPI, 4’,6-diamidino-2-phenylindole. Scale bar, 50 μm. **(E)** Cultured neurons were treated with 10 μM CORT alone or in combination with 0.1 μM fluoxetine or vehicle at 7 DIV for 72 h. Scale bar, 20 μm. **(F-G)** Representative immunoblots (left) and bar graph (right) showing nNOS ($F(3, 16) = 8.979$, CORT + vehicle versus vehicle: *$p = 0.0213$; CORT + FLX versus CORT + vehicle: ***$p < 0.0001$; CORT + FLX + Betulinic acid versus CORT + FLX: **$p = 0.0058$) and CAPON ($F(3, 16) = 12.22$, CORT + vehicle versus vehicle: **$p = 0.003$; CORT + FLX versus CORT + vehicle: ***$p < 0.001$; CORT + FLX + Betulinic acid versus CORT + FLX: **$p = 0.0017$) levels in the cultured neurons incubated with 10 μM

CORT alone or in combination with 0.1 μM fluoxetine and 20 μM Betulinic acid (n = 5 independent experiment). Data were normally distributed with Shapiro-Wilk test and one-way ANOVA followed by Tukey's multiple comparisons test **(A-C, F)** was carried out to test significance, *$P < 0.05$, **$P < 0.01$, ***$P < 0.001$. Graphs show Mean ± SEM. Veh: Vehicle; FLX: fluoxetine.

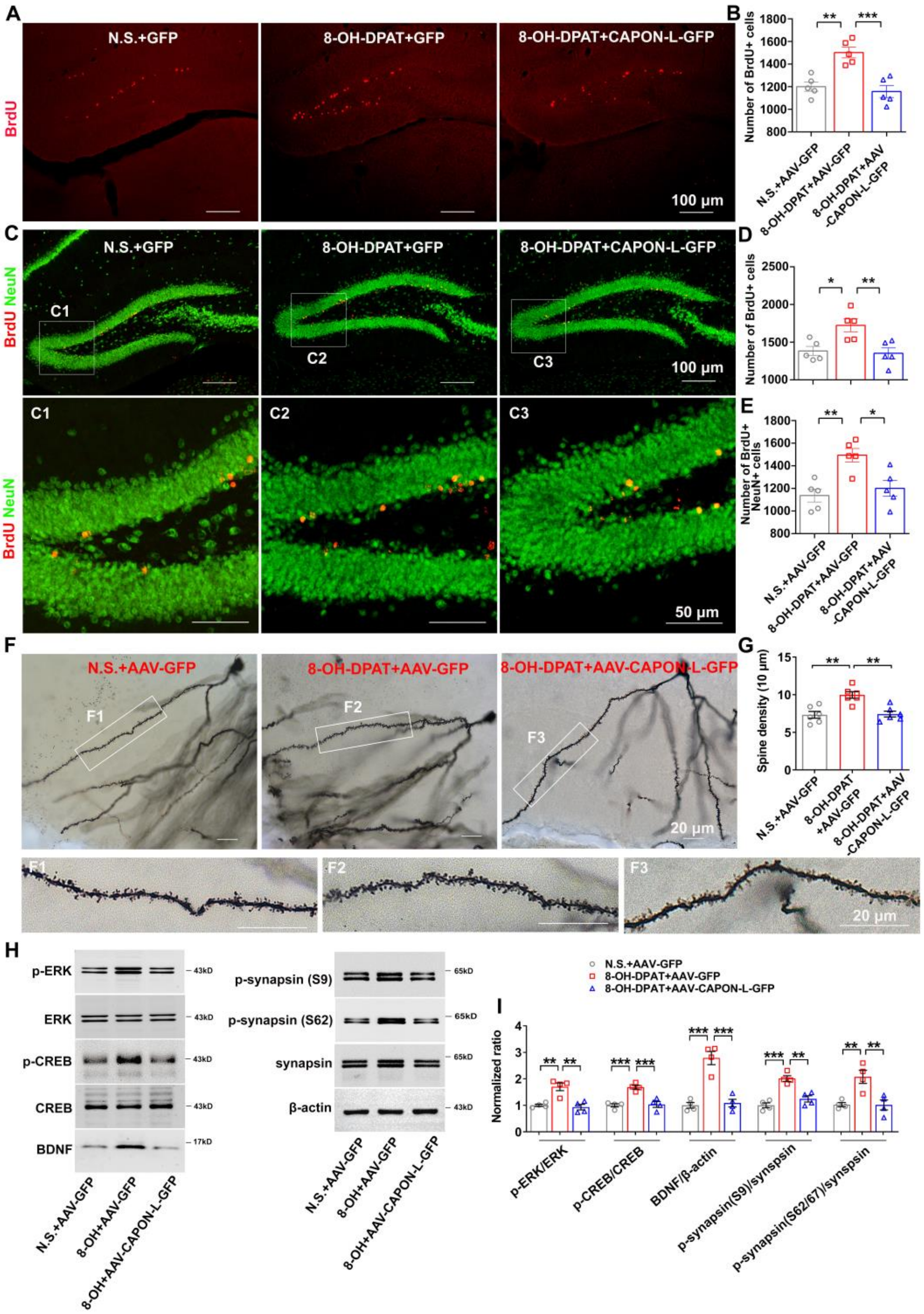

Figure S6 Augmenting nNOS-CAPON interaction reversed 5-HTAR agonist induced neurogenic and synaptogenic effects. (**A-I**) The adult mice treated with intra-hippocampal DG microinjection of AAV-CAPON-L-GFP or AAV-GFP in combination with 8-OH-DPAT (0.1 mg/kg/d i.p.) for 28 days. (**A-B**) Representative images (**A**) and bar graph (**B**) showing BrdU$^+$ cells (red) in the DG of mice exposed to

these treatments at 2 h after BrdU administration (n=5, $F(2,12) = 16.03$, AAV-GFP + 8-OH-DPAT versus AAV-GFP + vehicle: $^{**}p = 0.0013$; AAV-CAPON-L-GFP + 8-OH-DPAT versus AAV-GFP + 8-OH-DPAT: $^{***}p < 0.001$). Scale bar, 100 μm. (**C-E**) Representative images (**C**) and bar graph showing $BrdU^+$ cells (**D**) and $BrdU^+$/ $NeuU^+$ cells (**E**) in the DG of mice exposed to these treatments at 28 days after BrdU administration (n=5, for $BrdU^+$: $F(2,12) = 7.919$, AAV-GFP + 8-OH-DPAT versus AAV-GFP + vehicle: $^{*}p = 0.013$; AAV-CAPON-L-GFP + 8-OH-DPAT versus AAV-GFP + 8-OH-DPAT: $^{**}p = 0.0074$; for $BrdU^+$/ $NeuU^+$: $F(2,12) = 9.246$, AAV-GFP + 8-OH-DPAT versus AAV-GFP + vehicle: $^{**}p = 0.0033$; AAV-CAPON-L-GFP + 8-OH-DPAT versus AAV-GFP + 8-OH-DPAT: $^{*}p = 0.0123$). Scale bar, 100 μm, 50 μm. $BrdU^+$: red, $NeuU^+$: green. (**F-G**) Representative images with Golgi-Cox staining (**F**) and bar graph (**G**) showing dendrite spine density of granular cells in the hippocampal DG of mice exposed to different treatments (n = 6, 10 neurons per sample, $F(2,15) = 12.43$, AAV-GFP + 8-OH-DPAT versus AAV-GFP + vehicle: $^{**}p = 0.0014$; AAV-CAPON-L-GFP + 8-OH-DPAT versus AAV-GFP + 8-OH-DPAT: $^{**}p = 0.002$). Scale bar, 20 μm. (**H-I**) Representative immunoblots (**H**) and bar graph (**I**) showing p-ERK, p-CREB, p-synapsin, BDNF in the DG of mice with different treatments (n = 4, for p-ERK/ERK: $F(2,9) = 15.92$; AAV-GFP + 8-OH-DPAT versus AAV-GFP + vehicle: $^{**}p = 0.0031$; AAV-CAPON-L-GFP + 8-OH-DPAT versus AAV-GFP + 8-OH-DPAT: $^{**}p = 0.0017$; for p-CREB: $F(2,9) = 21.57$; AAV-GFP + 8-OH-DPAT versus AAV-GFP + vehicle: $^{***}p = 0.0007$; AAV-CAPON-L-GFP + 8-OH-DPAT versus AAV-GFP + 8-OH-DPAT: $^{***}p = 0.0009$;

for BDNF: $F(2,9) = 32.17$; AAV-GFP + 8-OH-DPAT versus AAV-GFP + vehicle: ***$p = 0.0001$; AAV-CAPON-L-GFP + 8-OH-DPAT versus AAV-GFP + 8-OH-DPAT: ***$p = 0.0002$; for p-syn(S9)/syn: $F(2,9) = 24.17$; AAV-GFP + 8-OH-DPAT versus AAV-GFP + vehicle: ***$p = 0.0002$; AAV-CAPON-L-GFP + 8-OH-DPAT versus AAV-GFP + 8-OH-DPAT: **$p = 0.0018$; for p-syn(S62/67)/syn: $F(2,9) = 10.85$; AAV-GFP + 8-OH-DPAT versus AAV-GFP + vehicle: **$p = 0.0077$; AAV-CAPON-L-GFP + 8-OH-DPAT versus AAV-GFP + 8-OH-DPAT: **$p = 0.0072$). Graphs show Mean ± SEM. Data were normally distributed with Shapiro-Wilk test and one-way ANOVA followed by Tukey's multiple comparisons test **(B, D, E, G, I)** was carried out to test significance, *$P < 0.05$, **$P < 0.01$, ***$P < 0.001$.
